# Protein in-cell NMR spectroscopy at 1.2 GHz

**DOI:** 10.1007/s10858-021-00358-w

**Published:** 2021-02-12

**Authors:** Enrico Luchinat, Letizia Barbieri, Matteo Cremonini, Lucia Banci

**Affiliations:** 1 Università degli Studi di Firenze, Via Luigi sacconi 6, 50019 Sesto Fiorentino, Italy; 2 Consorzio per lo Sviluppo dei Sistemi a Grande Interfase – CSGI, Via della Lastruccia 3, 50019 Sesto Fiorentino, Italy; 3 Consorzio Interuniversitario Risonanze Magnetiche di Metalloproteine, Via Luigi Sacconi 6, 50019 Sesto Fiorentino, Italy; 4 Dipartimento di Chimica, Università degli Studi di Firenze, Via della Lastruccia 3, 50019 Sesto Fiorentino, Italy

**Keywords:** In-cell NMR, Ultrahigh field NMR, TROSY, α-Synuclein, Cellular structural biology

## Abstract

In-cell NMR spectroscopy provides precious structural and functional information on biological macromolecules in their native cellular environment at atomic resolution. However, the intrinsic low sensitivity of NMR imposes a big limitation in the applicability of the methodology. In this respect, the recently developed commercial 1.2 GHz NMR spectrometer is expected to introduce significant benefits. However, cell samples may suffer from detrimental effects at ultrahigh fields, that must be carefully evaluated. Here we show the first in-cell NMR spectra recorded at 1.2 GHz on human cells, and we compare resolution and sensitivity against those obtained at 900 and 950 MHz. To evaluate the effects of different spin relaxation rates, SOFAST-HMQC and BEST-TROSY spectra were recorded on intracellular α-synuclein and carbonic anhydrase. Major improvements are observed at 1.2 GHz when analyzing unfolded proteins, such as α-synuclein, while the TROSY scheme improves the resolution for both globular and unfolded proteins.

**Supplementary Information:**

The online version contains supplementary material available at 10.1007/s10858-021-00358-w.

## Introduction

In-cell NMR is an application of biomolecular NMR spectroscopy to the characterization of the structure and dynamics of biological macromolecules inside living cells (Luchinat and Banci 2017; Siegal and Selenko 2019; Ito et al. 2020). This unique methodology relies on the high sensitivity of the chemical shift of the nuclear spins to the chemical surroundings to provide information at atomic resolution on the conformation, dynamics and interactions of a macromolecule in its physiologically relevant environment. In recent years, several examples of in-cell solution NMR both in bacterial and eukaryotic cells have demonstrated that, albeit challenging, the methodology can be applied to study protein structure (Sakakibara et al. 2009; Tanaka et al. 2019), conformation of nucleic acids (Dzatko et al. 2018; Broft et al. 2020), folding and maturation (Banci et al. 2013; Luchinat et al. 2014; Capper et al. 2018), effects of the environment on protein stability and compactness (Majumder et al. 2015; Smith et al. 2016; Theillet et al. 2016), chemical modifications (Binolfi et al. 2016; Mercatelli et al. 2016; Polykretis et al. 2019), and protein–drug interactions (DeMott et al. 2018; Luchinat et al. 2020a). Furthermore, time-resolved in-cell NMR is increasingly applied to observe intracellular events in real time through the use of NMR bioreactors to keep cells alive for longer periods of time (Sharaf et al. 2010; Kubo et al. 2013; Breindel et al. 2018; Cerofolini et al. 2019; Luchinat et al. 2020b).

Despite its huge potential, a major drawback of in-cell NMR compared to other cellular techniques is the low sensitivity. Such limitation derives from the intrinsic insensitivity of NMR spectroscopy, due to the small energy difference between the nuclear spin states compared to the thermal fluctuations at physiological temperatures, and is further exacerbated by the limited number of molecules of interest in the sample—with volume-averaged concentrations usually in the order of 5–500 µM—and by the short lifetime of the cells in the instrument. While sample stability can be improved to some extent, as shown in the aforementioned applications of bioreactor systems, the concentration of the molecule of interest cannot be increased above certain thresholds—which depend on the system under study—without decreasing the biological significance of the experiment. On the other hand, increasing the static magnetic field strength (B_0_) increases proportionally the energy splitting and the spin population difference, and increases the signal-to-noise ratio as a function of B_0_^3/2^, thus positively affecting both the resolution and the sensitivity of the technique.

Therefore, a major advancement in the applicability of in-cell NMR, and of NMR in general, is represented by the development of higher magnetic fields. The recent development of the first commercially available NMR spectrometer operating at 1.2 GHz was met with strong interest by the research community, and the advantages of using the new instrument for solution NMR experiments in vitro have already been shown (Banci et al. 2019). However, from those data it is not straightforward to assess how much benefit the new instrument would provide when analyzing macromolecules, such as proteins, in a cellular setting, as the theoretical improvements could be reduced by detrimental effects of the sample matrix, such as sample inhomogeneity, high ionic strength, and intracellular interactions.

In this work, we report the first protein NMR spectra recorded at 1.2 GHz on human cells expressing ^15^N-enriched proteins. Specifically, we focus on the 2D ^1^H–^15^N correlation spectra, the most commonly recorded experiments when studying proteins both in vitro and in cells, and provide a qualitative and quantitative comparison against two other high field NMR instruments, 900 and 950 MHz. The performance in terms of resolution and sensitivity was evaluated on an intrinsically disordered protein (IDP) and a globular protein, as they have very different relaxation properties that translate into different linewidths and heights of the NMR signals. Nuclear spin relaxation in a protein backbone is determined by dipole–dipole (DD) interactions and by the chemical shift anisotropy (CSA) (Pervushin et al. 1997). The extent to which these terms contribute to relaxation is dependent on local magnetic field fluctuations, which in turn depend on B_0_ and on the reorientation rate of the molecule. In the limit of rigid, globular proteins, the local motions of the backbone are negligible, and the transverse nuclear relaxation is determined by the rotational correlation time of the molecule, which increases with the molecular weight. Conversely, in the limit of a fully disordered polypeptide, such as that of an IDP, the nuclear relaxation is dominated by the local motions, and is independent of the molecular weight (Rezaei‐Ghaleh et al. 2012). Therefore, the performance of different spectrometers with increasing magnetic field strength was evaluated on samples of human cells overexpressing either human α-synuclein (α-Syn), an IDP with very little propensity for secondary structure formation (Wu et al. 2008), which has been extensively characterized by NMR both in bacterial and human cells (Waudby et al. 2013; Theillet et al. 2016), or the second isoform of human carbonic anhydrase (CA II), a stably folded globular protein (Krishnamurthy et al. 2008) that was recently shown to be free from interactions in the human cell cytoplasm and therefore amenable to in-cell NMR studies (Luchinat et al. 2020a, b).

Among the various pulse sequences available for ^1^H–^15^N correlation spectroscopy, SOFAST-HMQC and BEST-TROSY pulse sequences were selected for comparing the sensitivity and resolution of in-cell NMR experiments. Both sequences are optimized for increased sensitivity compared to the classical counterparts, using band-selective pulses for selective excitation of the ^1^H amide region, which results in shorter longitudinal relaxation and allows reducing the recycling delay. However, while the SOFAST-HMQC maximizes the overall sensitivity at the expense of resolution, the BEST-TROSY attains the highest resolution by selecting the TROSY component of the magnetization along both dimensions, at the expense of sensitivity. Therefore, the two sequences are at the extreme opposites when it comes to choose between resolution and sensitivity and can often provide complementary information.

## Materials and methods

### Gene cloning

To generate the mammalian expression plasmid, the cDNA encoding WT human α-Syn (amino acids 1–140, GenBank: NP_000336.1) was amplified by PCR from the pT7-7 asyn WT plasmid (Addgene, cat. #36046) and cloned into the pHLsec vector (Aricescu et al. 2006) between HindIII and XhoI restriction enzyme sites, following a previously reported cloning strategy (Barbieri et al. 2016). These restriction sites were chosen in order to remove a N-terminal signal peptide and a C-terminal histidine tag present in the original vector, so that the expression vector obtained with this strategy encodes the native protein sequences. The following primers were employed: forward: 5′-CCCAAGCTTGCCACCATGGATGTATTCATGAAAGGACTTTCAAAG-3′; reverse: 5′-CCGCTCGAGTTAGGCTTCAGGTTCGTAGTCTTG-3′. A Kozak sequence was inserted in the forward primer downstream of the HindIII site, while a stop codon was inserted in the reverse primers upstream of the XhoI site. The obtained vector (pHL-asyn) was verified by DNA sequencing. The empty plasmid, pHL-empty, and the plasmid containing the full-length WT human CA II gene (amino acids 1–260, GenBank: NP_000058.1), pHL-CAII, were already available in our laboratory and are described elsewhere (Banci et al. 2013; Luchinat et al. 2020a).

### Human cell cultures growth and transfection

HEK293T cells (ATCC CRL-3216) were maintained in Dulbecco-modified Eagle medium (DMEM) high glucose (Gibco) supplemented with l-glutamine, antibiotics (penicillin and streptomycin) and 10% fetal bovine serum (FBS, Gibco) in uncoated 75 cm^2^ plastic flasks and incubated at 37 °C, 5% CO_2_ in a humidified atmosphere. HEK293T cells were transiently transfected using branched polyethylenimine (PEI) following a previously reported protocol (Aricescu et al. 2006; Barbieri et al. 2016). Blank cell samples and cells expressing CA II were transfected with pHL-empty or pHL-CAII, respectively, mixed with PEI in a 1:2 ratio (25 μg/flask DNA + 50 μg/flask PEI). Cells expressing α-Syn were transfected with the following mixture: 5 μg/flask pHL-asyn + 20 μg/flask pHL-empty + 50 μg/flask PEI, in order to avoid cellular toxicity. Protein expression was carried out for 48 h in [U–^15^N]-BioExpress6000 medium (Cambridge Isotope Laboratories) supplemented with 2% FBS and antibiotics. For CA II expression, zinc was supplemented immediately after transfection as ZnSO_4_ to a final concentration of 10 μM in the expression medium.

### In-cell NMR sample preparation

Samples for in-cell NMR were prepared as previously reported (Barbieri et al. 2016). Briefly, transfected cells were detached with trypsin, suspended in DMEM + 10% FBS, washed once with PBS and re-suspended in one pellet volume of NMR medium, consisting of DMEM supplemented with 90 mM glucose, 70 mM HEPES and 20% D_2_O. The cell suspension was transferred in a 3 mm Shigemi NMR tube, which was gently spun to sediment the cells. Cell viability before and after NMR experiments was assessed by trypan blue exclusion assay. After the NMR experiments, the cells were collected and the supernatant was checked for protein leakage by NMR.

### Cell lysate sample preparation

Cell lysates were prepared from each cell sample by 8–10 freeze–thaw cycles in 1 pellet volume of PBS buffer, followed by centrifugation at 16,000×*g*, 4 °C for 1 h to remove the insoluble fraction. Averaged CA II and α-Syn cell lysates were prepared by pooling together the lysates from cells expressing CA II and those from cells expressing α-Syn, respectively. Three identical aliquots were placed in 3-mm Shigemi tubes and analyzed in parallel at the three instruments.

### NMR spectrometers and reference SNR

The following spectrometers were used in this study: Bruker Avance NEO 900 MHz (21.1 T) equipped with a 5 mm TCI Cryoprobe (basic transmitter frequency, BF1 = 900.35 MHz); Bruker Avance III 950 MHz (22.3 T) equipped with a 5 mm TCI Cryoprobe (BF1 = 950.2 MHz); Bruker Avance NEO 1.2 GHz (28.2 T) equipped with a 3 mm TCI Cryoprobe (BF1 = 1200.85 MHz). For comparison with the in-cell NMR samples, reference SNR values were measured on a 3-mm standard sample of 0.1% Ethylbenzene (EB) in CDCl_3_, giving 2988 at 900 MHz, 3028.1 at 950 MHz and 5274.1 at 1.2 GHz. Notably, the 3-mm reference SNR values obtained at 900 MHz and 950 MHz were lower than those obtained by rescaling the reference SNR values measured on a 5-mm standard sample of 0.1% EB in CDCl_3_ (10,256.8 at 900 MHz and 10,783 at 950 MHz) by the ratio of the inner sections of 3 mm (i.d. = 2.42 mm) and 5 mm (i.d. = 4.24 mm) NMR tubes (rescaled SNR values: 3341.3 at 900 MHz and 3512.7 at 950 MHz), due to the loss of efficiency of 5-mm probes in terms of SNR/number of spins when analyzing 3-mm samples.

### Setup of the NMR experiments

All NMR spectra were collected at 310 K. After ~ 3 min to allow uniform heating, the instruments were manually matched and tuned, shimming was done automatically with the command ‘topshim 1D shigemi’, and the 90° ^1^H pulse was calculated with the ‘pulsecal’ command. Cell and lysate samples were first checked by 1D ^1^H NMR (zgesgp, ~ 3 min acquisition) to assess the sample quality. 2D ^1^H–^15^N SOFAST-HMQC (sfhmqcf3gpph) and 2D ^1^H–^15^N BEST-TROSY (b_trosyf3gpph.2) spectra were subsequently recorded with the experimental parameters reported in Table S1. SOFAST-HMQC spectra were recorded with an interscan delay of 0.3 s, using the shaped pulses Pc9_4_90.1000 and Reburp.1000 for selective ^1^H inversion and refocusing, respectively. BEST-TROSY spectra were recorded with an interscan delay of 0.25 s, using the shaped pulses Eburp2.1000/Eburp2tr.1000 and Reburp.1000 for selective ^1^H inversion and refocusing, respectively. Shaped pulse lengths and power levels were automatically calculated (‘-DCALC_SP’ option). For α-Syn, excitation width and offset were set to 4 and 8.3 ppm, respectively; for CA II they were set to 5.4 and 8.7 ppm, respectively.

### Processing and analysis of the spectra

2D NMR spectra were processed in Topspin by applying a square sine bell window function (SSB = 2) and zero filling in both dimensions. Linear prediction (LPmifc, 64 coefficients) was applied along the ^15^N dimension before Fourier transform. To better resolve signals in the central region of the spectra, each processed spectrum was copied and further processed by subtracting a second NMR spectrum recorded with identical parameters on a sample of cells transfected with pHL-empty vector, in order to remove the background signal arising from non-specific cellular ^15^N incorporation (Figs. S1 and S2). Sequence-specific resonance assignments were retrieved from BMRB entry 16543 for α-Syn (Bodner et al. 2010) and BMRB entry 34308 for CA II (Singh et al. 2019). Spectral resolution was measured on three isolated peaks (see the Results section) by extracting the row (for ^1^H) and column (for ^15^N) 1D traces at the corresponding chemical shift (‘slice’ command in Topspin). Full-width at half maximum (FWHM) values were obtained with the ‘peakw’ command. SNR was calculated on the ^1^H 1D trace of each peak with the ‘sino’ command by manually setting the signal and noise regions for each trace. Spectral sensitivity (SNR_t_) was calculated with the following formula: SNR_t_ = SNR/(t^1/2^), where t = total experimental time (Lee et al. 2014). For α-Syn, resolution was measured on the background-subtracted spectra to avoid errors in the FWHM measurement caused by the overlap with the ^15^N-background envelope, whereas sensitivity was measured on the non-subtracted spectra; for CA II, both resolution and sensitivity were measured on the non-subtracted spectra.

## Results

For the comparison of in-cell NMR spectra, samples of human cells overexpressing either α-Syn or CA II uniformly enriched with ^15^N were analyzed at 900 MHz, 950 MHz, and 1.2 GHz. Fresh cell samples were prepared for the analysis at each spectrometer to ensure that the NMR spectra were recorded on viable cells. For each sample, both SOFAST-HMQC and BEST-TROSY spectra were recorded. The experiments were set up to allow proper comparison across the three spectrometers: identical spectral windows and acquisition times in both direct and indirect dimensions were chosen (Table S1, see also “Materials and methods”), therefore providing the same spectral resolution (given the same transverse relaxation rate). Shaped pulses excitation windows (in ppm) and processing parameters were also kept constant (Table S1). The number of scans was adjusted to keep similar total experimental times ≲ 2 h, to minimize possible differences arising from sample changes over time and to preserve cell viability (Barbieri et al. 2016). A similar set of NMR spectra was also recorded on two ‘averaged’ cell lysates, which were obtained by pooling together the lysates of all the α-Syn-expressing cells and all the CA II-expressing cells, respectively. Each ‘averaged’ lysate was divided in three identical aliquots to be analyzed at each spectrometer (see “Materials and methods”). The acquisition parameters were optimized to account for the higher resolution of lysate NMR compared to in-cell NMR (Table S1). This experimental setup averages out the sample-to-sample variability of protein expression, allowing a more accurate comparison of the sensitivity of the three spectrometers.

**Fig. 1:**
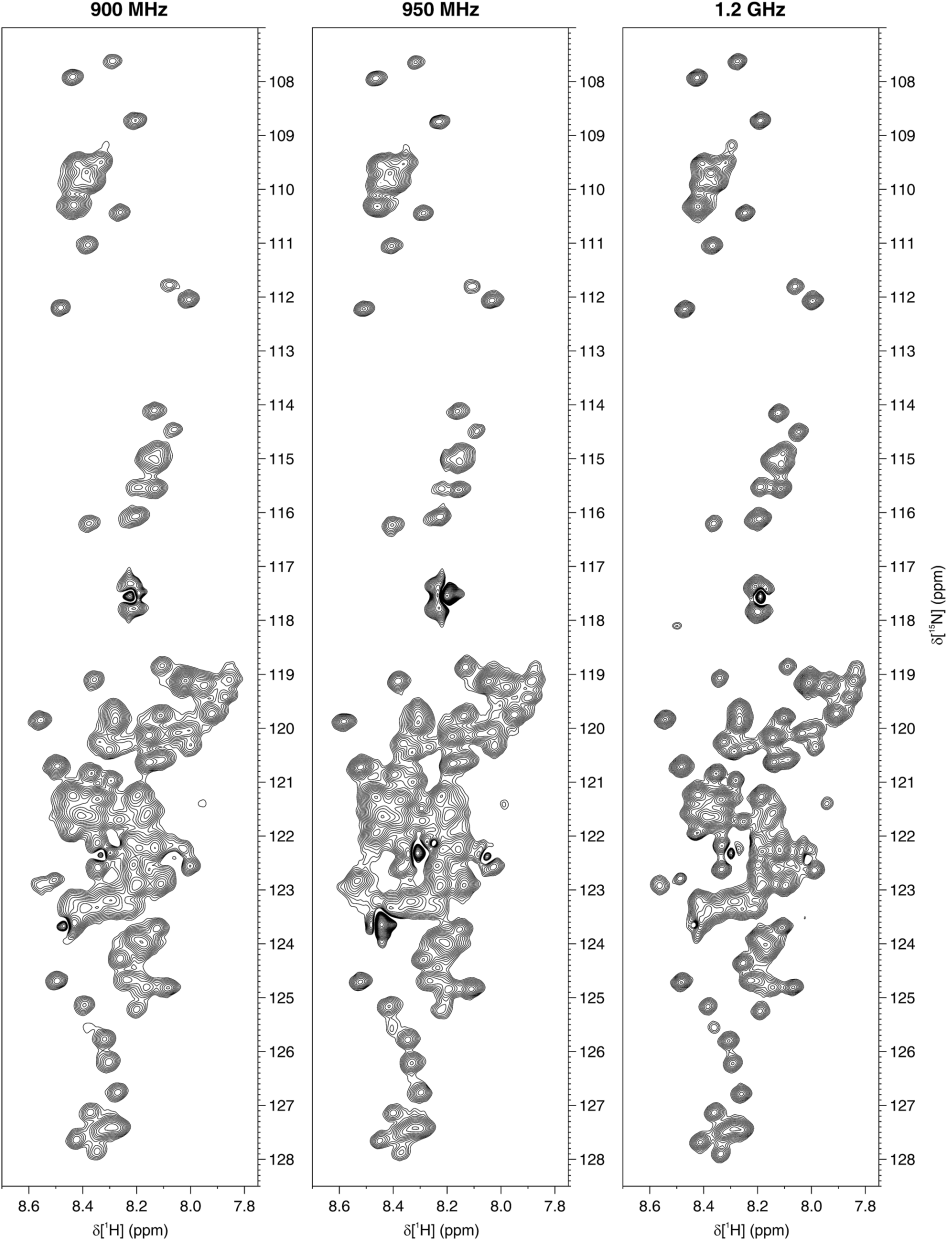
^1^H–^15^N SOFAST-HMQC recorded at 900 MHz (left), 950 MHz (center) and 1.2 GHz (right) on cells expressing α-Syn. The spectra were background-subtracted (see “Materials and methods”). The lowest contour level was set to 4 × the noise height

**Fig. 2:**
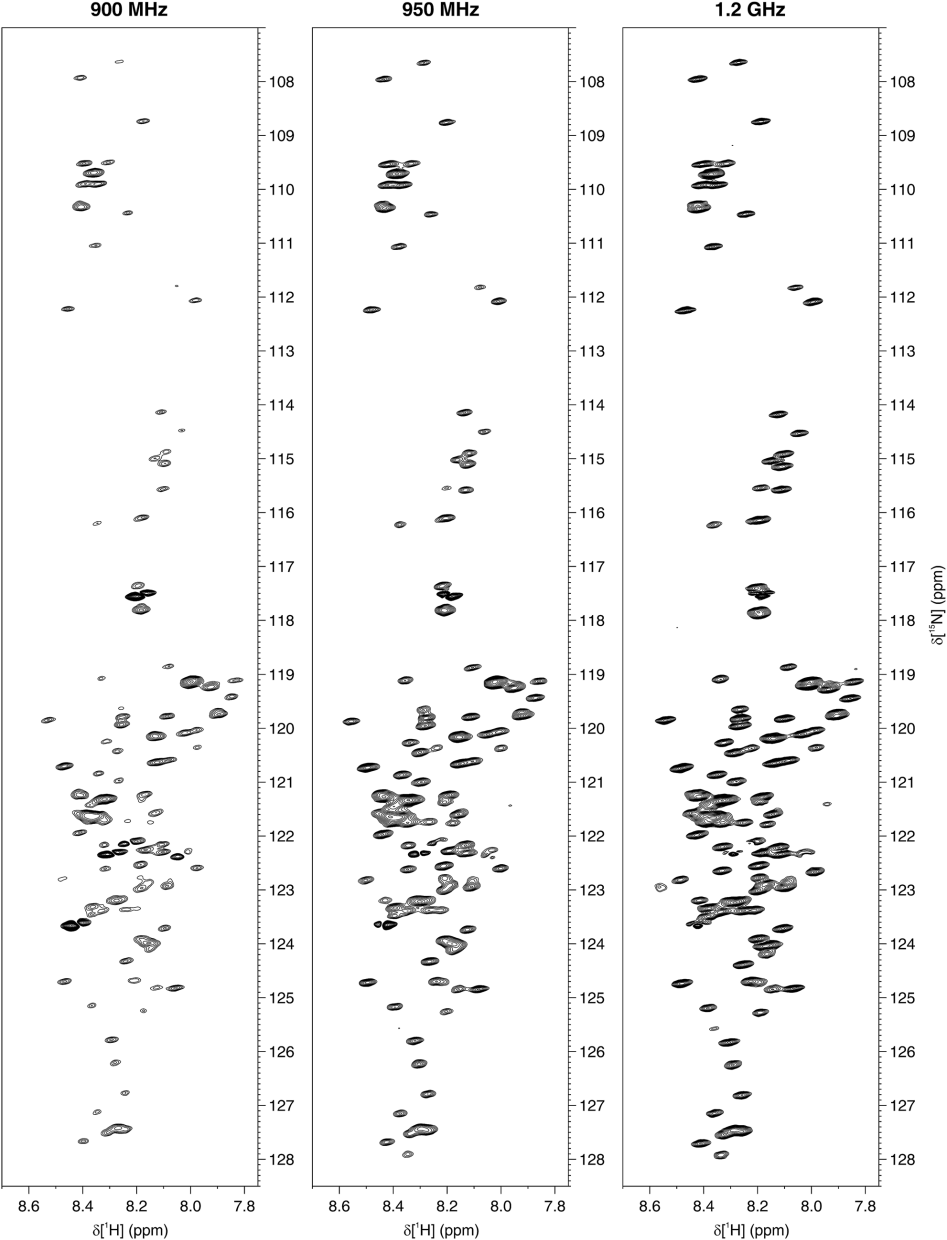
^1^H–^15^N BEST-TROSY recorded at 900 MHz (left), 950 MHz (center) and 1.2 GHz (right) on cells expressing α-Syn. The spectra were background-subtracted (see “Materials and methods”). The lowest contour level was set to 4 × the noise height

**Fig. 3:**
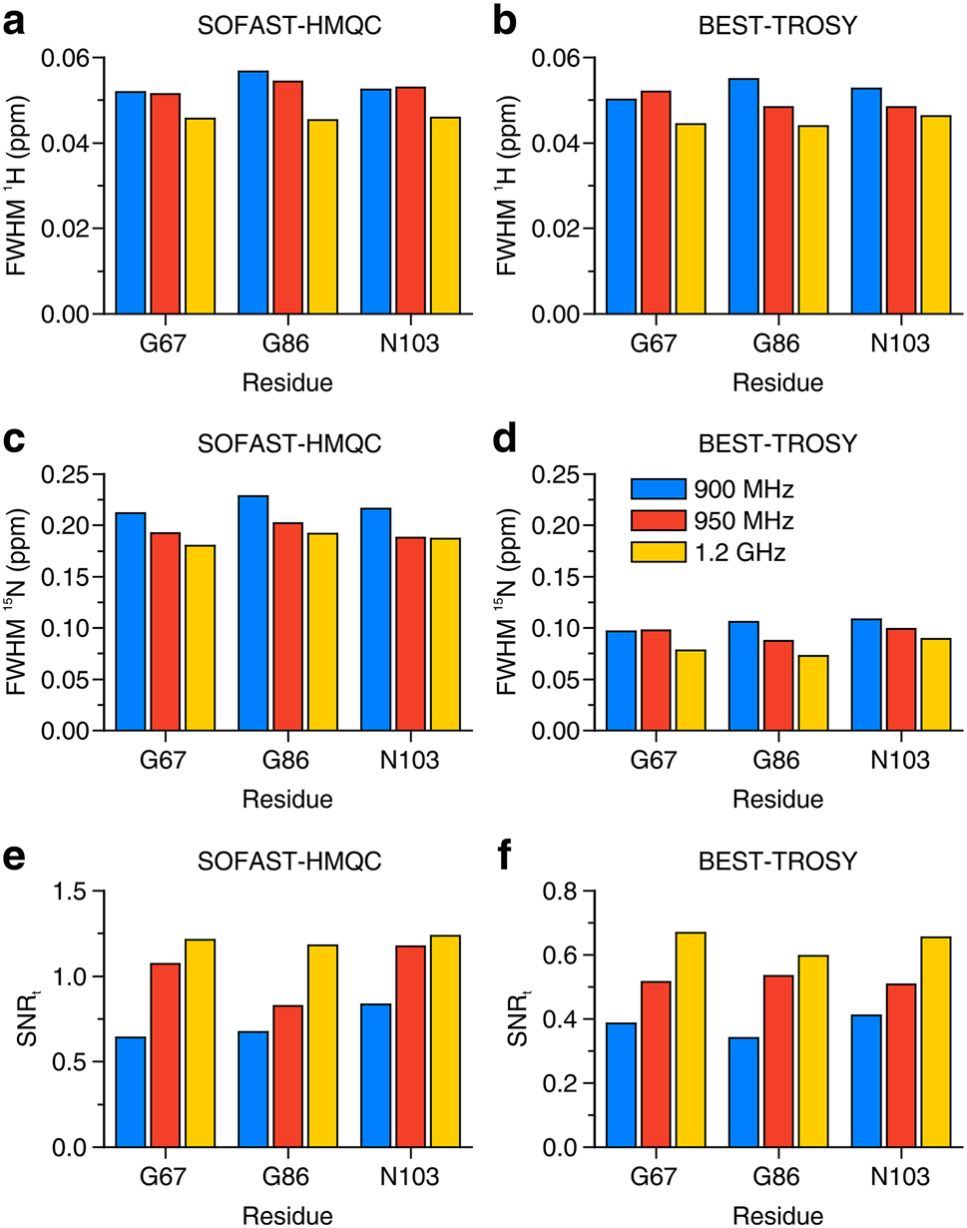
Resolution and sensitivity of α-Syn in-cell NMR spectra. **a**–**d** Spectral resolution of SOFAST-HMQC (**a**, **c**) and BEST-TROSY (**b**, **d**) spectra along the ^1^H (**a**, **b**) and the ^15^N (**c**, **d**) dimension, reported as FWHM (ppm) for three peaks at each spectrometer. **e**, **f** Sensitivity calculated as SNR_t_ = SNR/(t^1/2^) for each peak in the SOFAST-HMQC (**e**) and in the BEST-TROSY (**f**) spectra. Blue: 900 MHz; red: 950 MHz; yellow: 1.2 GHz

**Fig. 4:**
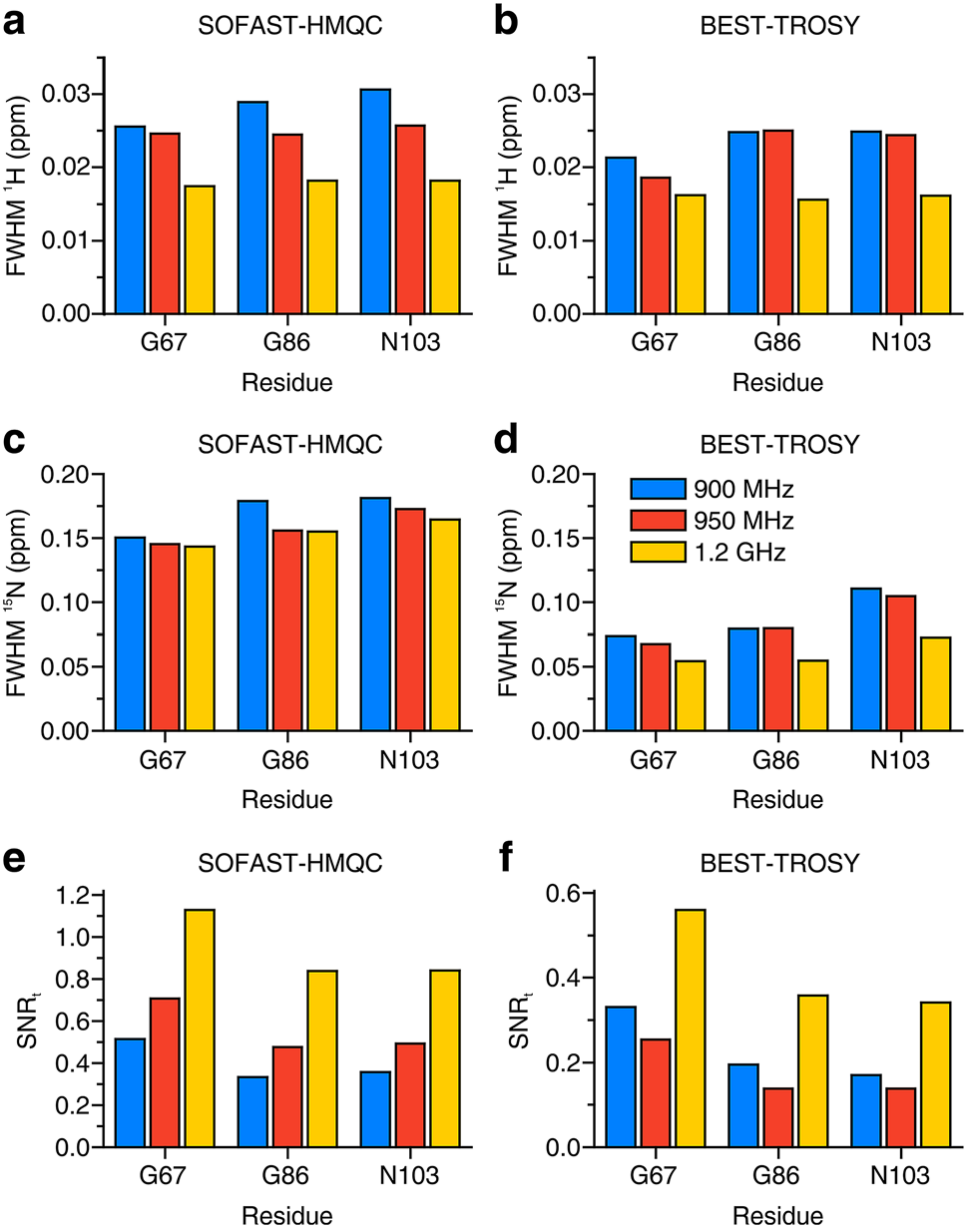
Resolution and sensitivity of the NMR spectra of the α-Syn cell lysate. **a**–**d** Spectral resolution of SOFAST-HMQC (**a**, **c**) and BEST-TROSY (**b**, **d**) spectra along the ^1^H (**a**, **b**) and the ^15^N (**c**, **d**) dimension, reported as FWHM (ppm) for three peaks at each spectrometer. **e**, **f** Sensitivity calculated as SNR_t_ = SNR/(t^1/2^) for each peak in the SOFAST-HMQC (**e**) and in the BEST-TROSY (**f**) spectra. Blue: 900 MHz; red: 950 MHz; yellow: 1.2 GHz

The in-cell NMR spectra of α-Syn are shown in Figs. 1 and 2. While all three instruments recorded high-quality in-cell NMR spectra, the increased resolution at increasing B_0_ can be appreciated. For resolution and sensitivity analysis, well-resolved signals from G67, G86 and N103 were chosen (Fig. S3), mainly because of the weak cellular background signal at their position (Fig. S1). Quantitative analysis shows clear improvements both in resolution and sensitivity at increasing magnetic fields (Fig. 3). The resolution improved in both dimensions of the SOFAST-HMQC and BEST-TROSY spectra (Fig. 3a–d). The highest increase in resolution was in the ^15^N dimension of the BEST-TROSY spectrum (the linewidth of G86 expressed in ppm decreased by ~ 31% from 900 MHz to 1.2 GHz, Fig. 3d). The sensitivity of α-Syn in-cell NMR markedly increased from 900 MHz to 1.2 GHz, with improvements of SNR_t_ up to ~ 90% in the SOFAST-HMQC and ~ 75% in the BEST-TROSY (Fig. 3e, f). Notably, the SNR_t_ improvements varied considerably for different residues and did not follow the same trend with increasing B_0_, likely due to the intrinsic uncertainty when measuring low SNR values. Similar results were observed in the α-Syn cell lysate NMR spectra (Figs. S4–S6). Compared to those of intact cells, the NMR spectra of the cell lysate reached a much higher resolution at all spectrometers, as expected from the fact that, unlike intact cells, lysates do not suffer from intrinsic inhomogeneity of the sample, which causes non-homogeneous line broadening. Quantitative analysis on the lysate NMR spectra confirmed a clear improvement of both resolution and sensitivity with a more consistent trend at increasing B_0_ (Fig. 4). For the BEST-TROSY, we noted that the 950 MHz was systematically the least sensitive, possibly due to different hardware and pulse power levels calibration (Fig. 4f).

**Fig. 5:**
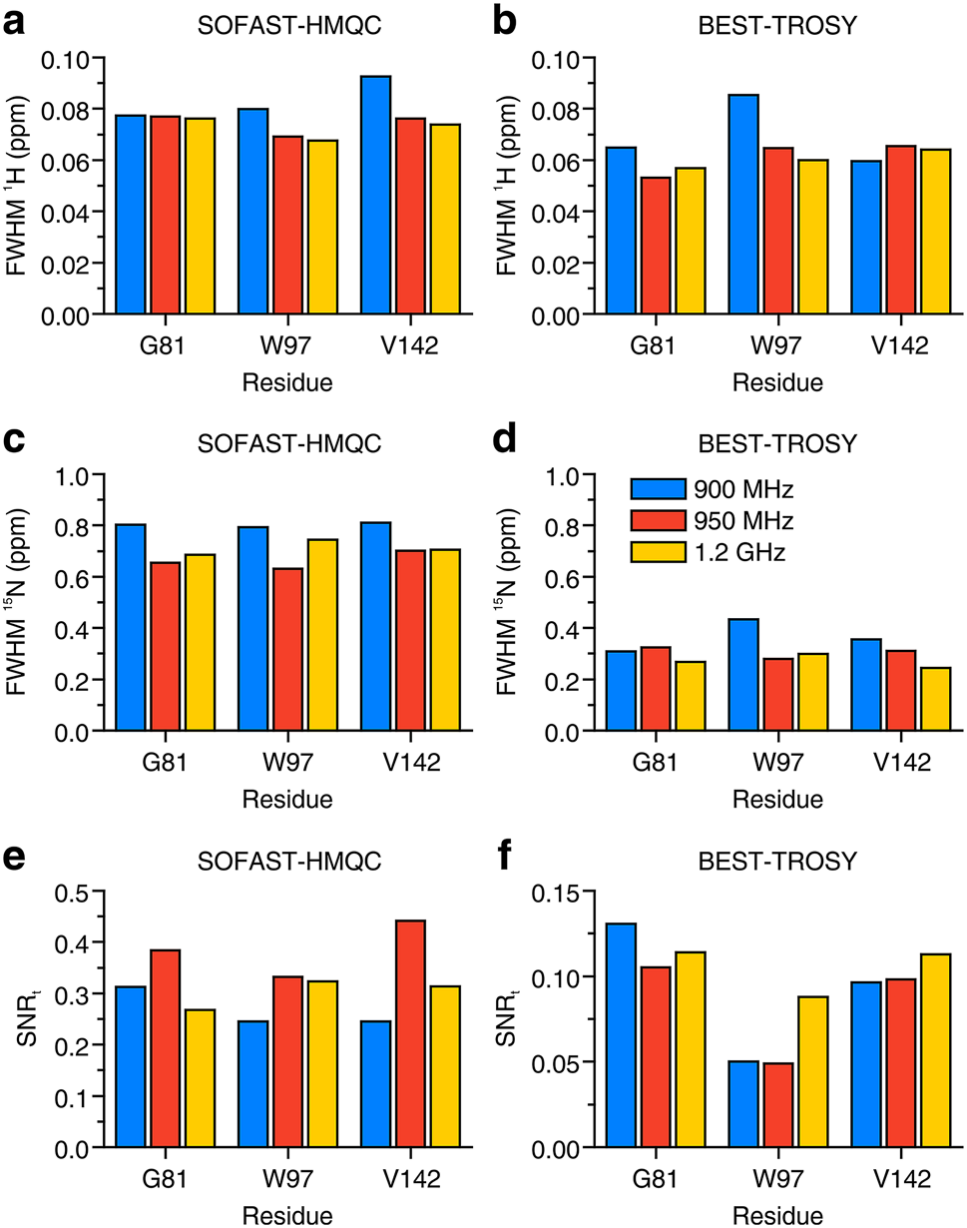
Resolution and sensitivity of CA II in-cell NMR spectra. **a**–**d** Spectral resolution of SOFAST-HMQC (**a**, **c**) and BEST-TROSY (**b**, **d**) spectra along the ^1^H (**a**, **b**) and the ^15^N (**c**, **d**) dimension, reported as FWHM (ppm) for three peaks at each spectrometer. (**e**, **f**) Sensitivity calculated as SNR_t_ = SNR/(t^1/2^) for each peak in the SOFAST-HMQC (**e**) and in the BEST-TROSY (**f**) spectra. Blue: 900 MHz; red: 950 MHz; yellow: 1.2 GHz

**Fig. 6:**
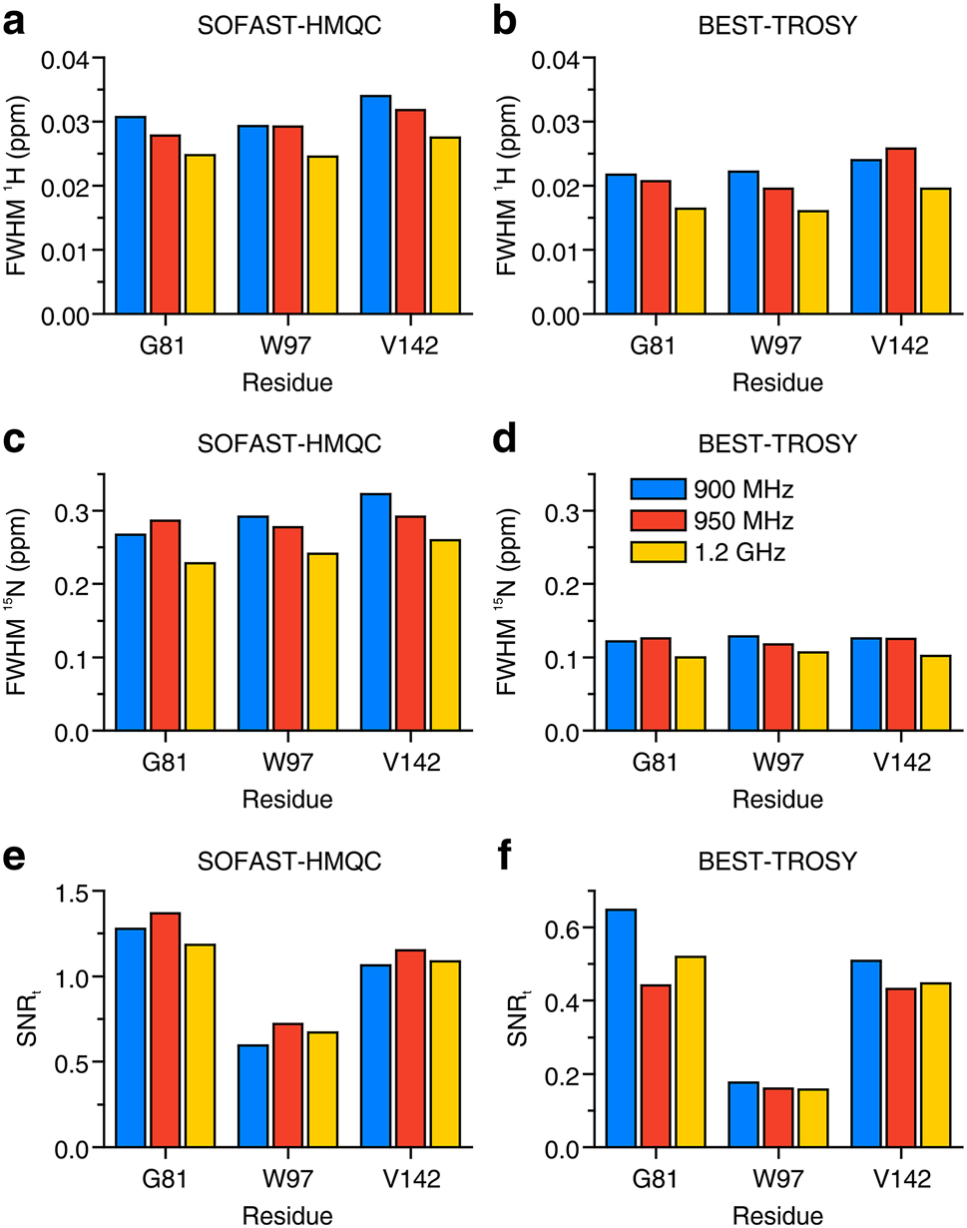
Resolution and sensitivity of the NMR spectra of the CA II cell lysate. **a**–**d** Spectral resolution of SOFAST-HMQC (**a**, **c**) and BEST-TROSY (**b**, **d**) spectra along the ^1^H (**a**, **b**) and the ^15^N (**c**, **d**) dimension, reported as FWHM (ppm) for three peaks at each spectrometer. **e**, **f** Sensitivity calculated as SNR_t_ = SNR/(t^1/2^) for each peak in the SOFAST-HMQC (**e**) and in the BEST-TROSY (**f**) spectra. Blue: 900 MHz; red: 950 MHz; yellow: 1.2 GHz

The benefits of a higher magnetic field for in-cell NMR were somewhat less evident in the case of CA II (Figs. S7 and S8). Signals from G81, W97 and V142 were chosen for quantitative analysis of spectral resolution and sensitivity, as they are well resolved and located in different regions of the spectrum (Fig. S9), far from the cellular background signal envelope (Fig. S2). W97 and V142 are located in rigid structural elements of CAII (β-sheets), while G81 lies on the protein surface and likely experiences a higher flexibility. Quantitative analysis of CA II in-cell NMR spectra (Fig. 5) revealed minor improvements in the resolution of both experiments, however with large variability between signals (Fig. 5a–d). A clearer improvement was still observed in the BEST-TROSY ^15^N dimension, with ~ 31% increased resolution of W97 and V142 from 900 MHz to 1.2 GHz (Fig. 5d), similar to what was observed for α-Syn. Conversely, no clear trend was observed when comparing sensitivity (Fig. 5e, f), likely due to increasing uncertainty when estimating low SNR values, and also possibly as a consequence of small variations in the expression levels of CA II in the three samples. The same analysis was carried out on the NMR spectra of CA II cell lysate (Figs. S10–S12), and resulted in a much clearer increase of resolution of ^1^H and ^15^N in both types of experiments (Fig. 6a–d). However, still no clear trend was observed in the sensitivity of the SOFAST-HMQC, (Fig. 6e), whereas a loss of sensitivity of the BEST-TROSY was observed at increasing magnetic field (Fig. 6f).

## Discussion and conclusions

The ^1^H–^15^N correlation spectra analyzed above were collected at different spectrometers on samples of cells which were obtained following the same protein expression protocol, which is highly reproducible, and on identical fractions of the pooled-together cell lysates. Therefore, analysis of a set of representative signals allowed a straight up comparison of the performance of the different instruments, both in terms of resolution and sensitivity, for in-cell NMR applications to unfolded (α-Syn) and folded (CA II) proteins. In addition to the effect of the increasing magnetic field strength, hardware differences between the spectrometers also affected the final outcome of the NMR experiments. Therefore, the comparison shown here may not be immediately related to what expected from theoretical considerations. In general, the spectral quality improved at higher magnetic fields for both proteins, although the extent of the improvement varied considerably.

In the spectra of α-Syn, resolution and sensitivity increased with a clear trend from 900 MHz to 1.2 GHz, both in the cells (Figs. 1, 2 and S3) and in the lysate (Figs. S4–S6). As expected, the benefits of higher fields when studying IDPs are evident, as the increased frequency separation between the signals results in a higher resolution, which is critical to reduce spectral overlap in the highly crowded spectra of unfolded proteins. Remarkably, we even observed a slight decrease in the apparent ^1^H linewidths of α-Syn in the cell lysate from 900 MHz to 1.2 GHz (Fig. S13). This suggests that a minor contribution to the resolution gain at 1.2 GHz may come from a decrease in ^1^H transverse relaxation rates. Whether this is really the case for α-Syn and/or other IDPs at ultra-high magnetic fields, and which are the implications for the dynamics of IDPs in solution is worth further investigation beyond this work.

Furthermore, we observed a marked increase in sensitivity, up to a factor of ~ 2.3 between 900 MHz and 1.2 GHz and a factor of ~ 1.7 between 950 MHz and 1.2 GHz (values obtained from SOFAST-HMQC spectra on the cell lysate, see Fig. 4e). A ~ 2-fold increase in sensitivity is an obvious advantage for preserving cell viability during in-cell NMR experiments, as it translates in a ~ 4-fold experimental time reduction for a given protein concentration. The ~ 1.7 increase in sensitivity observed from 950 MHz to 1.2 GHz is consistent with the reference SNR values obtained on a 3-mm standard sample of 0.1% ethylbenzene (see “Materials and methods”), which increases by a factor of 1.74, whereas the observed increase from 900 MHz to 1.2 GHz is even higher (~ 2.3 compared to 1.77), suggesting that differences in hardware and/or shaped pulse calibration between the 900 MHz and the 950 MHz could affect the SOFAST-HMQC performance more than that of the 90° hard pulse used to measure the standard sample SNR. It should be noted that for all samples the sensitivity is always higher than the theoretical increase in SNR, which is proportional to B_0_^3/2^ × T_2_ and would result in a signal height increase of a factor of 1.54 (from 900 MHz to 1.2 GHz) and 1.42 (from 950 MHz to 1.2 GHz), neglecting the dependence of T_2_ on B_0_ (Takeuchi et al. 2016). This is a consequence of the decreased performance of 3-mm NMR tubes in a 5-mm probe compared to that of the same 3-mm NMR tubes in a 3-mm probe (see “Materials and methods” for a comparison between SNR values measured on 5-mm and 3-mm reference samples).

In the case of CA II, minor differences were observed between different spectrometers, that did not show a clear trend as a function of field strength (e.g., the SOFAST-HMQC has maximum sensitivity at 950 MHz, Fig. 5e), or were not consistent among different residues (see for example the ^1^H linewidth of W97 and V142 in the BEST-TROSY, Fig. 5b). The latter effect may arise from the uncertainty caused by the low SNR of the CA II spectra, while the former could be caused by differences in the electronics of the AVANCE NEO (900 MHz and 1.2 GHz) and the AVANCE III (950 MHz), which may affect the shaped pulse performance. Complex protein dynamics could also affect differently the transverse relaxation of the investigated residues, impacting both resolution and sensitivity. Nevertheless, a measurable increase in resolution along the ^15^N dimension of the BEST-TROSY was still observed at 1.2 GHz (Fig. 5d). The higher resolution attained at 1.2 GHz can be also appreciated from the separation of the E26 and S129 crosspeaks (Fig. S9). In the lysate sample, both the SOFAST-HMQC and the BEST-TROSY were more resolved at 1.2 GHz (Fig. 4a–d and S12). The gain in resolution in both the HMQC and the TROSY might be surprising when considering that the line broadening in a folded protein increases due to the faster CSA-driven transverse relaxation, and that the theoretical maximum of the T_2_ of the TROSY component is reached at lower fields (~ 900 to 1000 MHz) (Pervushin et al. 1997; Takeuchi et al. 2015, 2016). Our observation implies that, in a folded protein, the increase in signal separation at higher fields overcomes the line broadening induced by the faster transverse relaxation, resulting in a higher resolution of both the TROSY and the non-TROSY components of the NMR spectrum.

Interestingly, however, the resolution in cells did not increase as much as in the lysates, for both proteins. This sort of ‘ceiling effect’ of the in-cell NMR spectral resolution is especially evident along the ^1^H dimension of the BEST-TROSY (Figs. 1, S3, S8 and S9). Most likely, this effect is caused by inhomogeneous line broadening induced by magnetic field inhomogeneities within the cell pellet. These inhomogeneities, which could be caused by the presence of µm- to mm-thick layers of materials with different magnetic susceptibilities (buffer, membranes, cytosol etc.), cannot be compensated by the shimming coils, and their effect would increase proportionally with the magnetic field, thus counterbalancing the increased signal separation with additional line broadening. Because of the different gyromagnetic ratios, the effect is more deleterious for ^1^H than for ^15^N, as observed in the spectra. The same phenomenon would also explain the elongation “along the diagonal” of the crosspeaks in the in-cell BEST-TROSY spectra (Figs. S3 and S9), that is totally absent in the NMR spectra of the lysates (Figs. S5, S6, S11 and S12).

The obvious benefits of higher magnetic fields for solution NMR spectroscopy must be carefully evaluated for different systems under study. Biological macromolecules at physiological temperature can undergo internal and global motions that span a wide range of amplitudes and rates, resulting in very different spin relaxation properties. When they are investigated in living cells, additional effects from the sample make even harder to predict how much, if at all, a higher field will improve increased resolution and sensitivity. The above analysis shows the practical benefits of the first operating 1.2 GHz spectrometer when performing in-cell solution NMR experiments on IDPs and globular proteins. While both protein types gave rise to qualitatively good ^1^H–^15^N in-cell NMR spectra, quantitative comparison revealed a striking improvement in sensitivity and resolution for IDPs. Conversely, no clear improvements in sensitivity were observed when analyzing globular proteins, and the obtained values were more affected by inter-residue variability and hardware differences. However, the BEST-TROSY experiment improved the spectral resolution also for the folded protein, indicating that the selection of slow-relaxing components is still beneficial, despite relaxation minimum of the TROSY components being reached at lower fields, without sacrificing sensitivity, which reaches the theoretical maximum at 1.2 GHz (Takeuchi et al. 2016). Finally, a considerable increase in inhomogeneous line broadening was observed, as a consequence of the small-scale inhomogeneity of the cell pellet in the NMR tube. This detrimental effect may be mitigated—although at the cost of a decreased signal intensity—by suspending cells at reduced density in hydrogels such as methylcellulose, which greatly improve sample homogeneity and have been recently employed to record high resolution NMR spectra of small metabolites and an IDP interacting with cell receptors (Alshamleh et al. 2020; Mateos et al. 2020).

## Supplementary Information

Below is the link to the electronic supplementary material.

Supplementary file1 (DOCX 25781 KB)

## Data Availability

The datasets generated and/or analyzed during the current study are available from the corresponding author on reasonable request.
