# Supplementary material for: Protein in-cell NMR spectroscopy at 1.2 GHz
Source: J Biomol NMR. 2021 Feb 12;75(2):97–107. doi: 10.1007/s10858-021-00358-w (PMC8018933; doi:10.1007/s10858-021-00358-w)
Supplement: Supplementary file 1 — Supplementary file1 (DOCX 25781 KB) [file 10858_2021_358_MOESM1_ESM.docx]

Supplementary Information

**Protein in-cell NMR spectroscopy at 1.2 GHz**

Enrico Luchinat^1,2,*^, Letizia Barbieri^1,3^, Matteo Cremonini^1^, Lucia Banci^1,4,*^

^1^ CERM – Magnetic Resonance Center, Università degli Studi di Firenze, Via Luigi sacconi 6, 50019 Sesto Fiorentino, Italy;

^2^ Consorzio per lo Sviluppo dei Sistemi a Grande Interfase – CSGI, Via della Lastruccia 3, 50019 Sesto Fiorentino, Italy;

^3^ Consorzio Interuniversitario Risonanze Magnetiche di Metalloproteine, Via Luigi Sacconi 6, Sesto Fiorentino, Italy;

^4^ Dipartimento di Chimica, Università degli Studi di Firenze, Via della Lastruccia 3, 50019 Sesto Fiorentino, Italy;

Enrico Luchinat: eluchinat@cerm.unifi.it;

Lucia Banci: banci@cerm.unifi.it

**Contents**

Figures S1-S13 2

Table S1 15

**
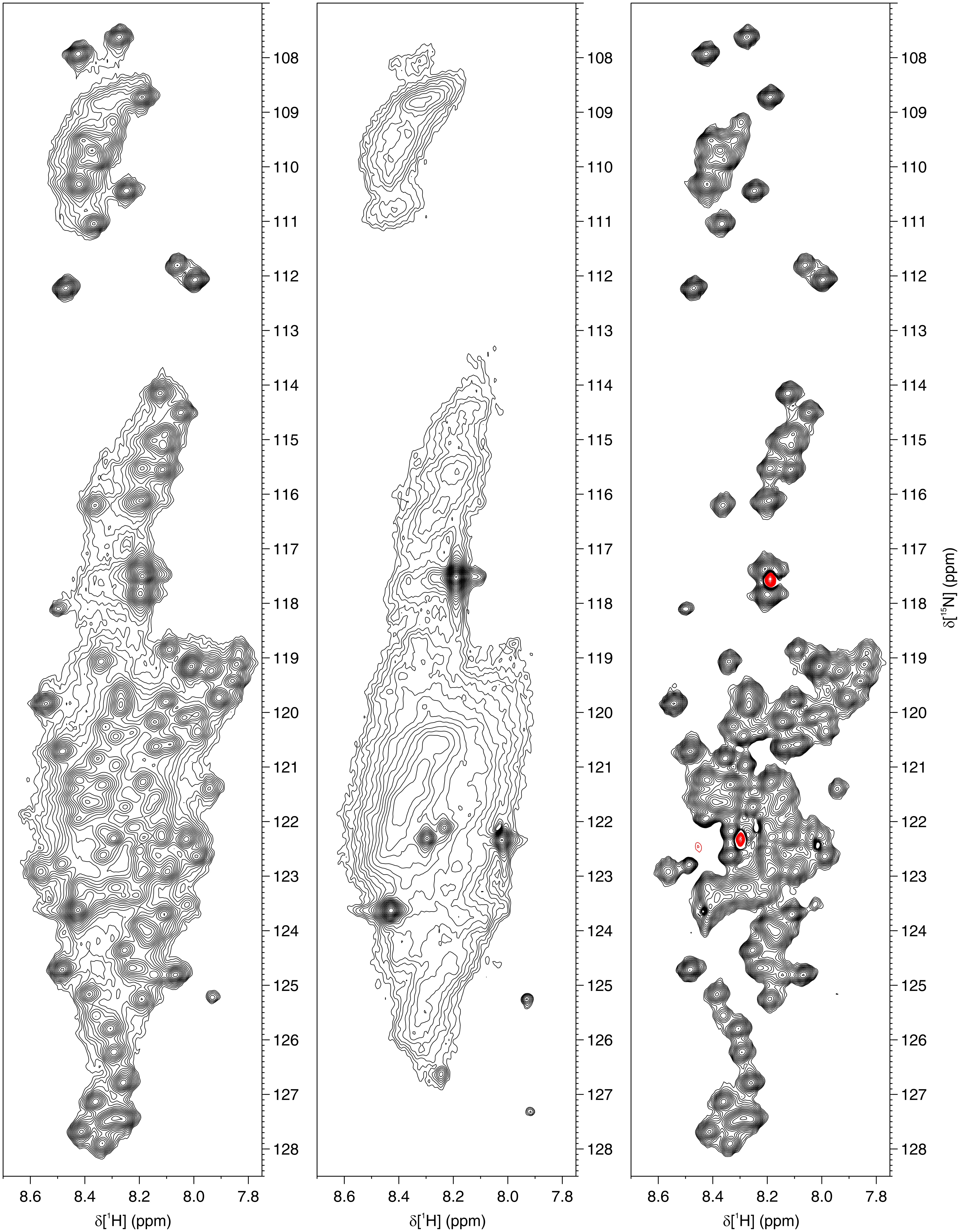
**

**Fig. S1** Raw ^1^H-^15^N SOFAST-HMQC spectra recorded at 1.2 GHz on cells expressing α-Syn in ^15^N-labeled medium for 48 h (left) and on cells transfected with an empty vector incubated in ^15^N-labeled medium for 48 h (center). Subtraction of the latter from the former results in a clean spectrum of intracellular α-Syn (right). Small negative peaks (red) result from minor differences in the cellular background envelope, and were omitted for clarity in the other figures.


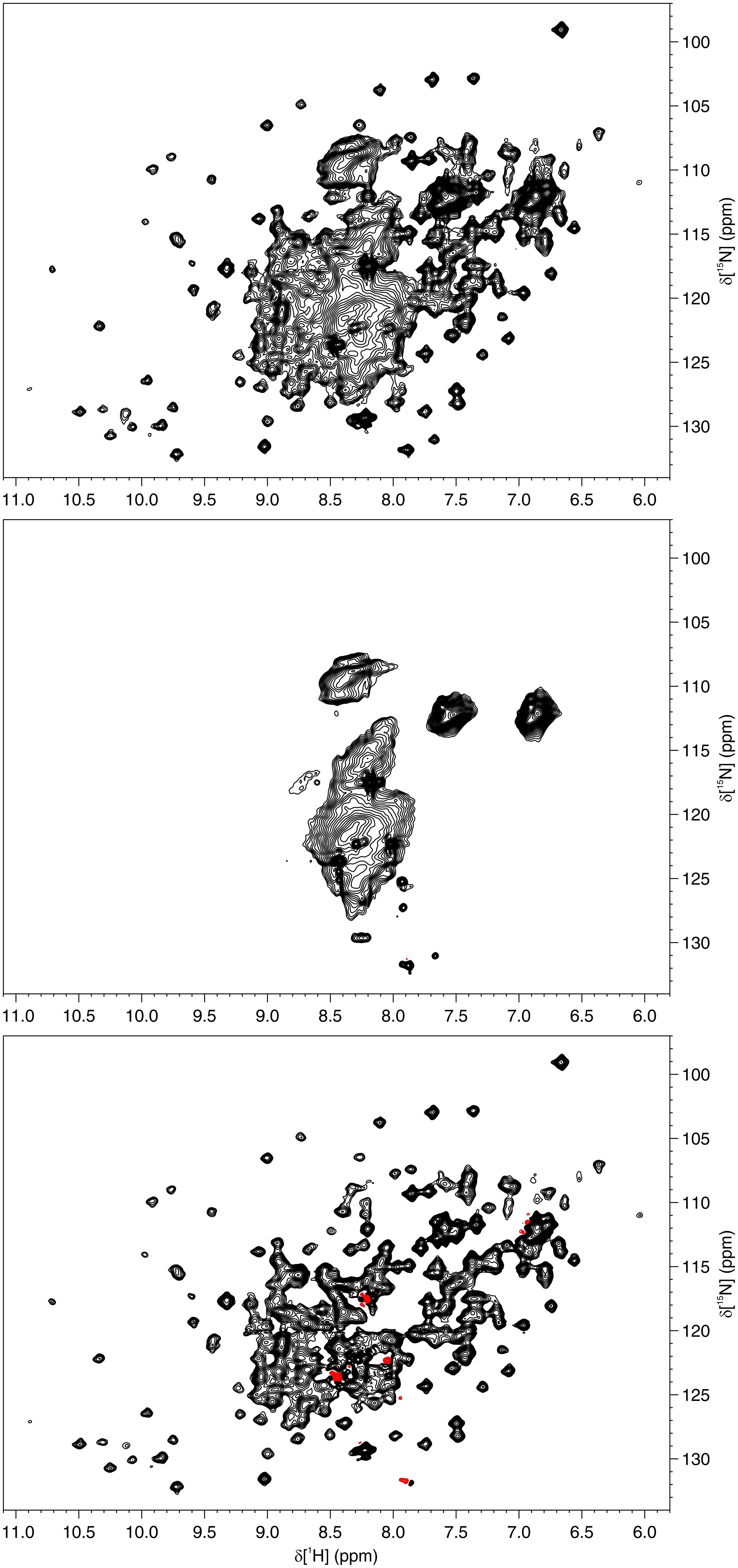


**Fig. S2** ^1^H-^15^N SOFAST-HMQC spectra recorded at 1.2 GHz on cells expressing CA II in ^15^N-labeled medium for 48 h (top) and on cells transfected with an empty vector incubated in ^15^N-labeled medium for 48 h (middle). Subtraction of the latter from the former results in a clean spectrum of intracellular CA II (bottom). Small negative peaks (red) result from minor differences in the cellular background envelope, and were omitted for clarity in the other figures.

**
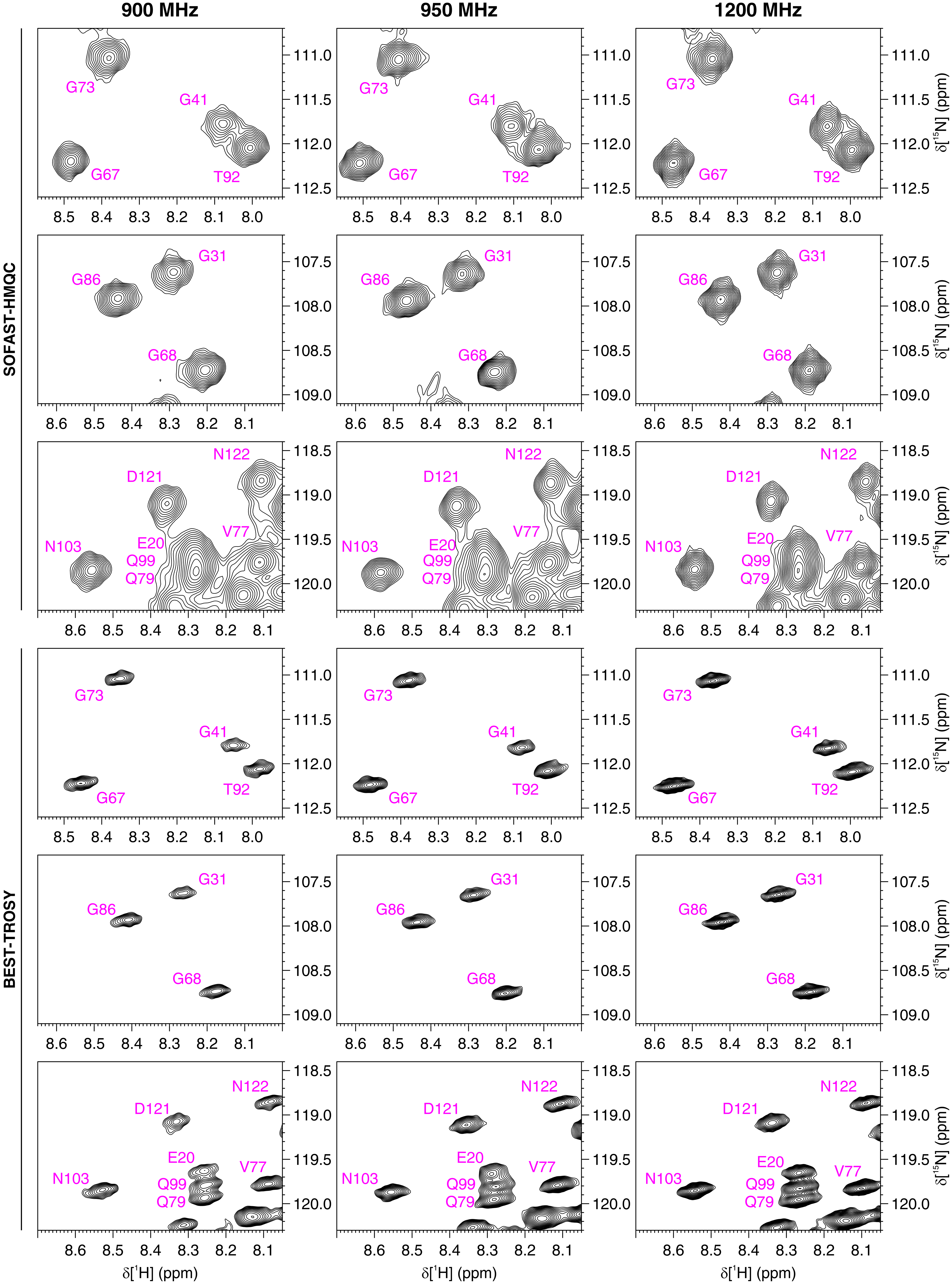
**

**Fig. S3** Spectral regions of ^1^H-^15^N SOFAST-HMQC (top series) and ^1^H-^15^N BEST-TROSY (bottom series) recorded at 900 MHz (left), 950 MHz (center) and 1.2 GHz (right) on cells expressing α-Syn. Spectra were background-subtracted (see Materials and Methods) and the lowest contour level was set to 2x the noise height. Amide crosspeaks are labeled according to their residues (magenta).


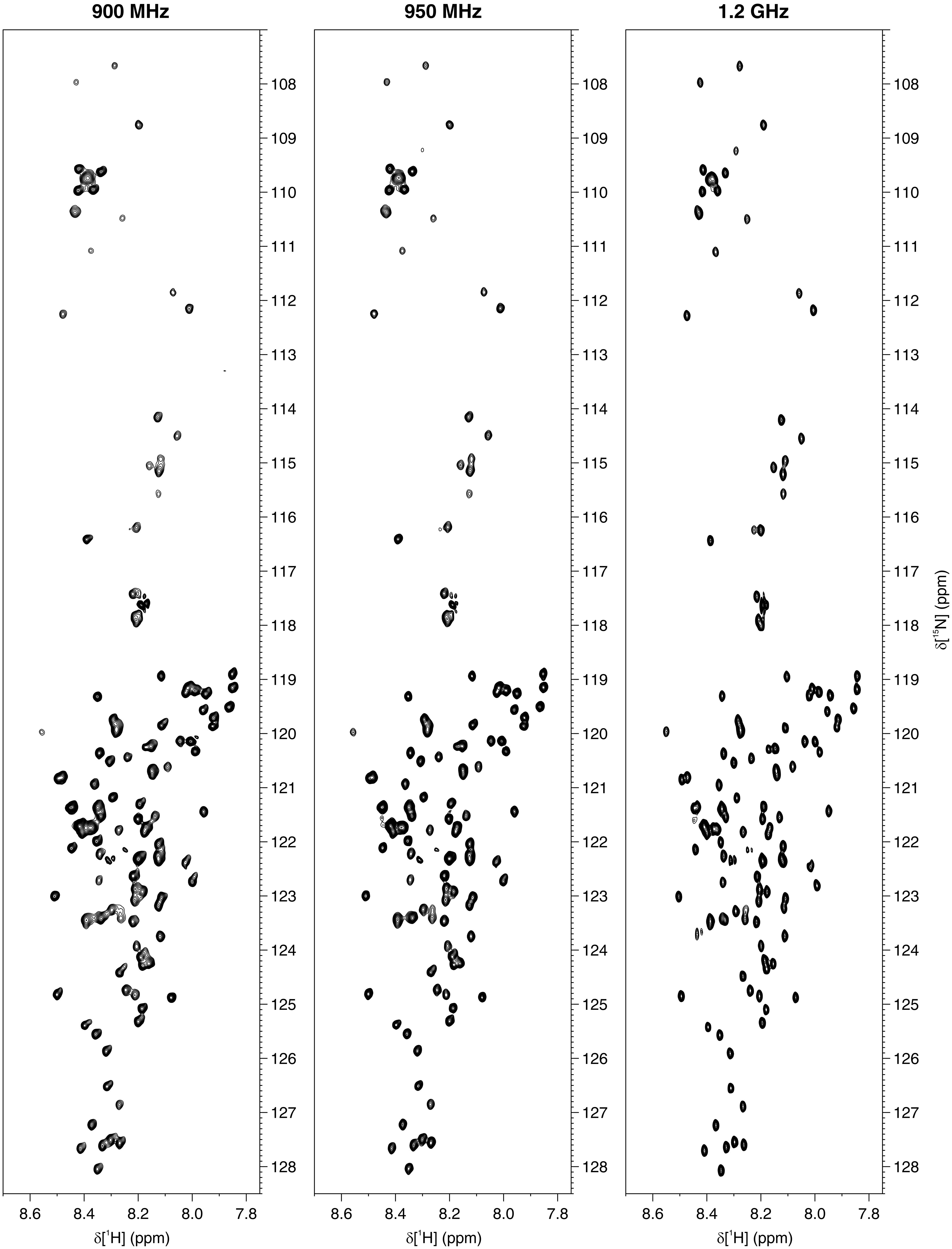


**Fig. S4** ^1^H-^15^N SOFAST-HMQC recorded at 900 MHz (left), 950 MHz (center) and 1.2 GHz (right) on the lysate from cells expressing α-Syn. The spectra were background-subtracted (see Materials and Methods). The lowest contour level was set to 4x the noise height.


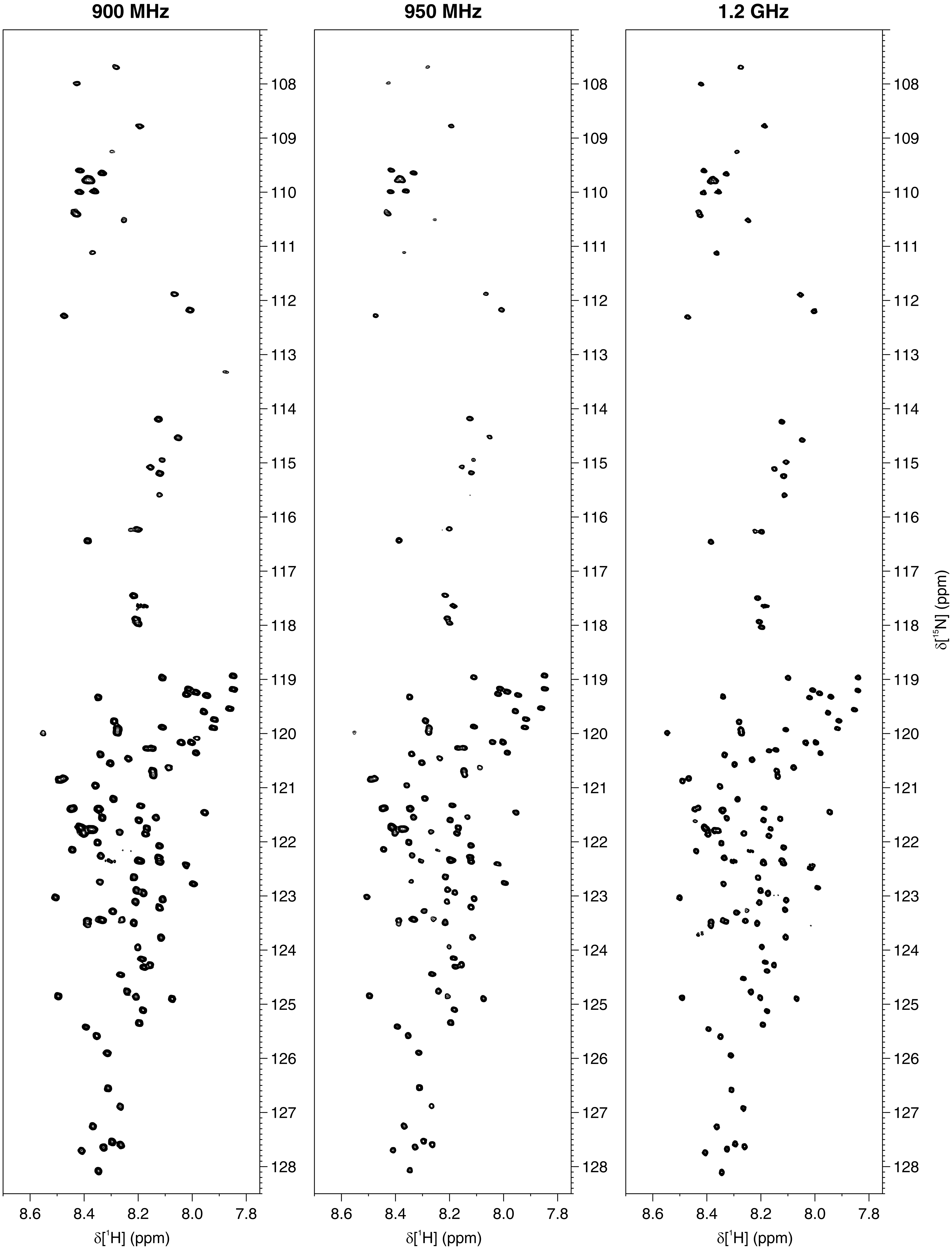


**Fig. S5** ^1^H-^15^N BEST-TROSY recorded at 900 MHz (left), 950 MHz (center) and 1.2 GHz (right) on the lysate from cells expressing α-Syn. The spectra were background-subtracted (see Materials and Methods). The lowest contour level was set to 2x the noise height.


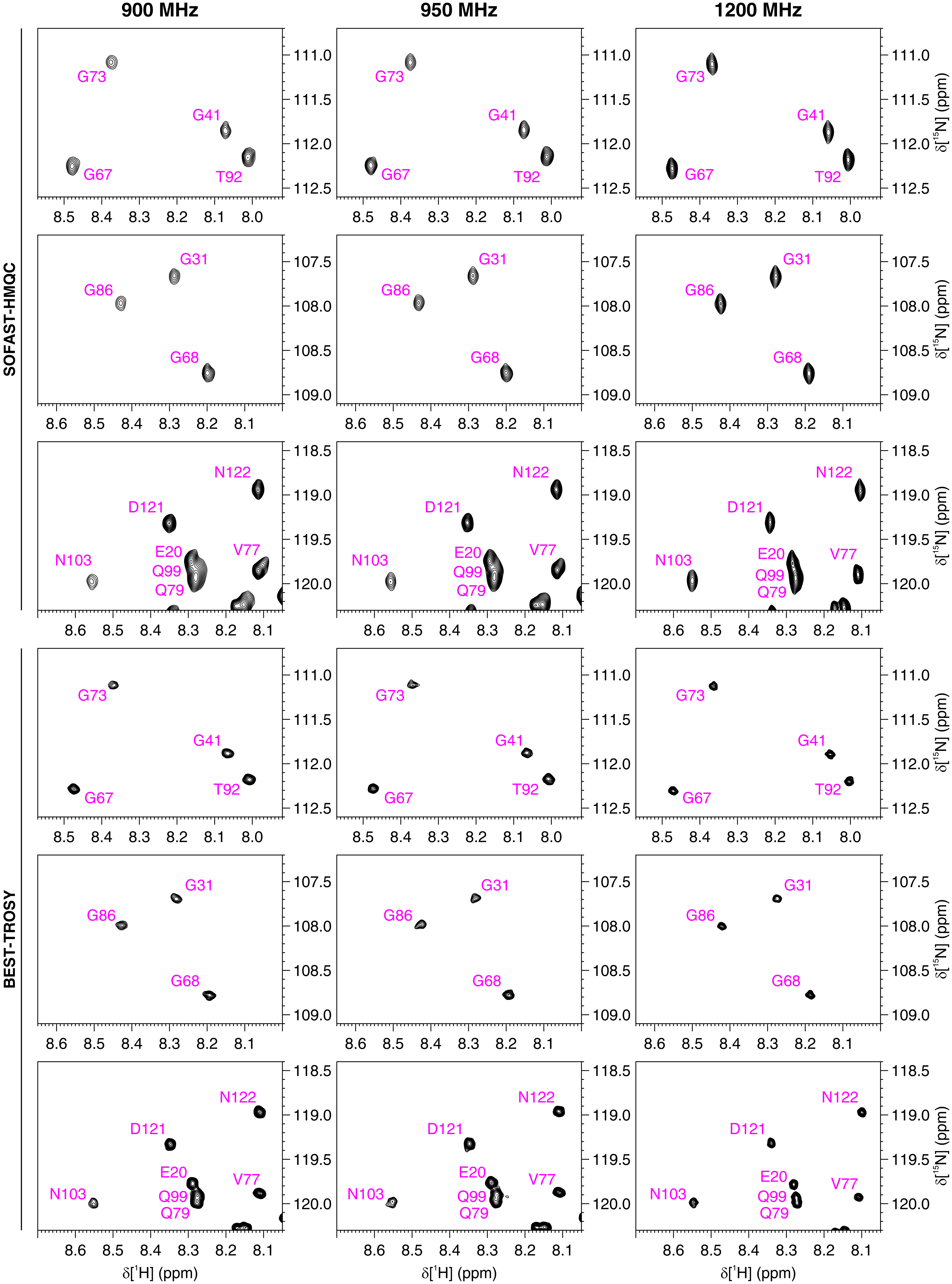


**Fig. S6** Spectral regions of ^1^H-^15^N SOFAST-HMQC (top series) and ^1^H-^15^N BEST-TROSY (bottom series) recorded at 900 MHz (left), 950 MHz (center) and 1.2 GHz (right) on the lysate from cells expressing α-Syn. Spectra were background-subtracted (see Materials and Methods) and the lowest contour level was set to 2x the noise height. Amide crosspeaks are labeled according to their residues (magenta).


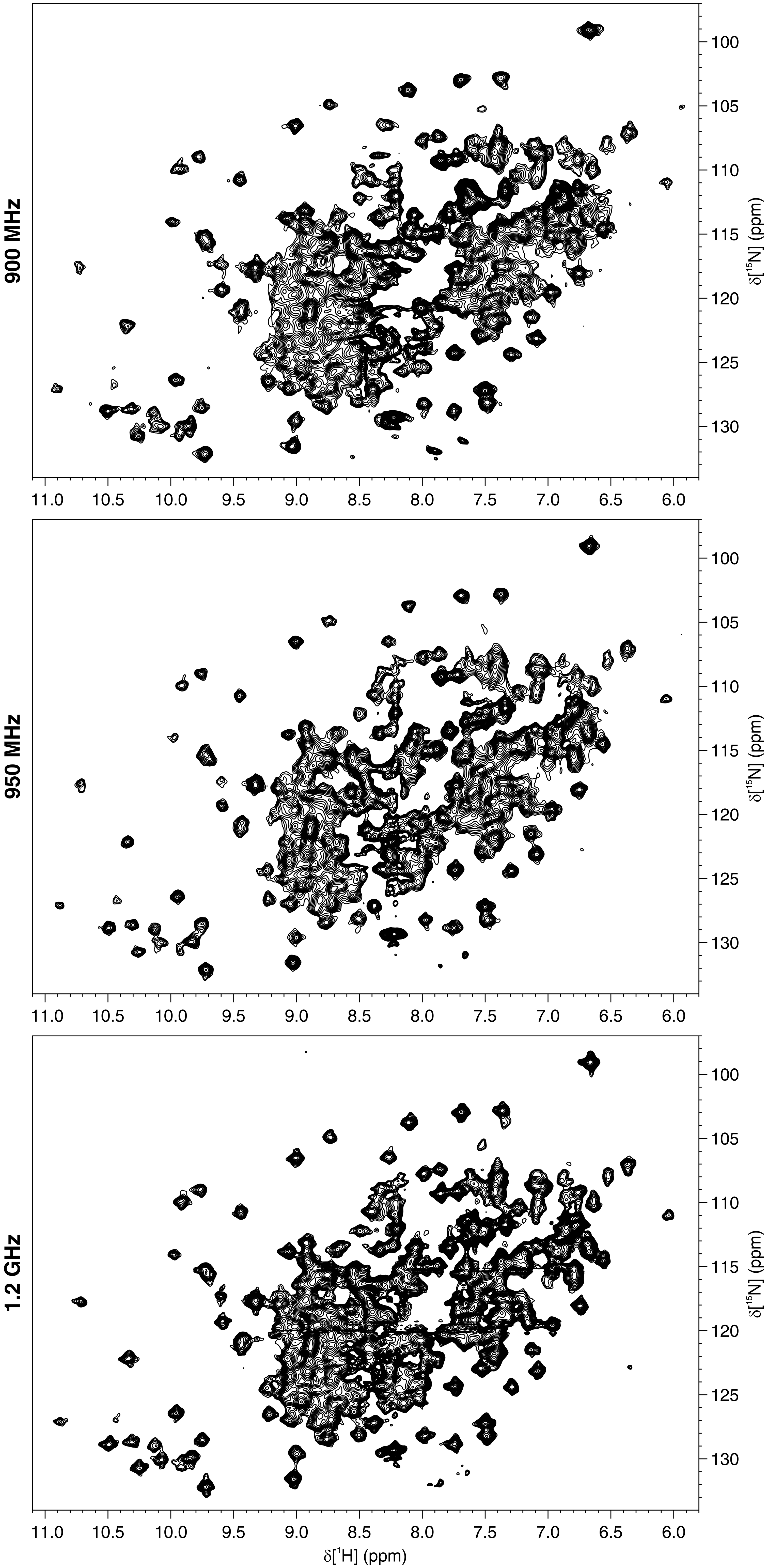


**Fig. S7** ^1^H-^15^N SOFAST-HMQC recorded at 900 MHz (top), 950 MHz (middle) and 1.2 GHz (bottom) on cells expressing CA II.
The spectra were background-subtracted (see Materials and Methods). The lowest contour level was set to the noise height.


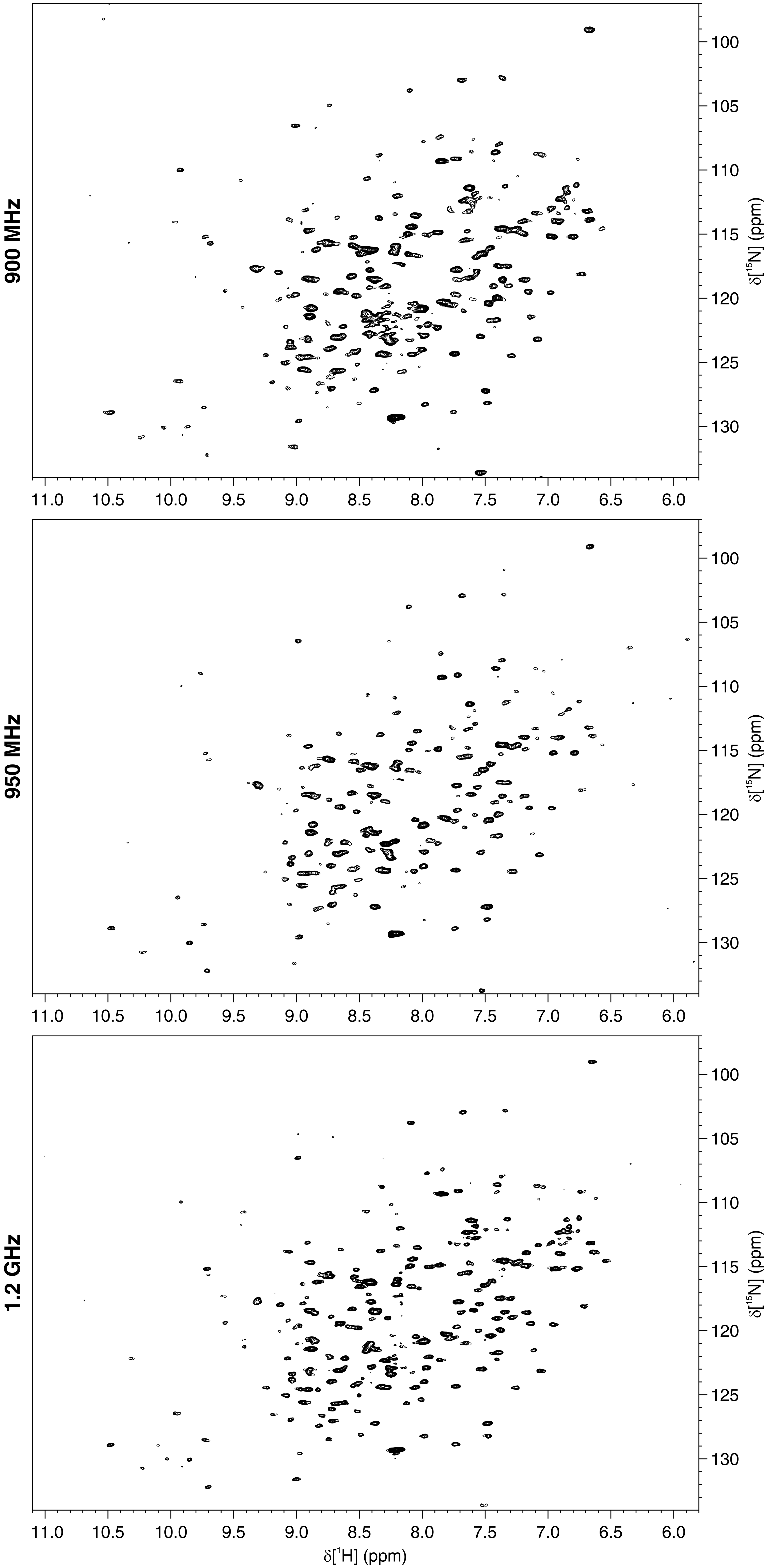


**Fig. S8** ^1^H-^15^N BEST-TROSY recorded at 900 MHz (top), 950 MHz (middle) and 1.2 GHz (bottom) on cells expressing CA II.
The spectra were background-subtracted (see Materials and Methods). The lowest contour level was set to the noise height.

**
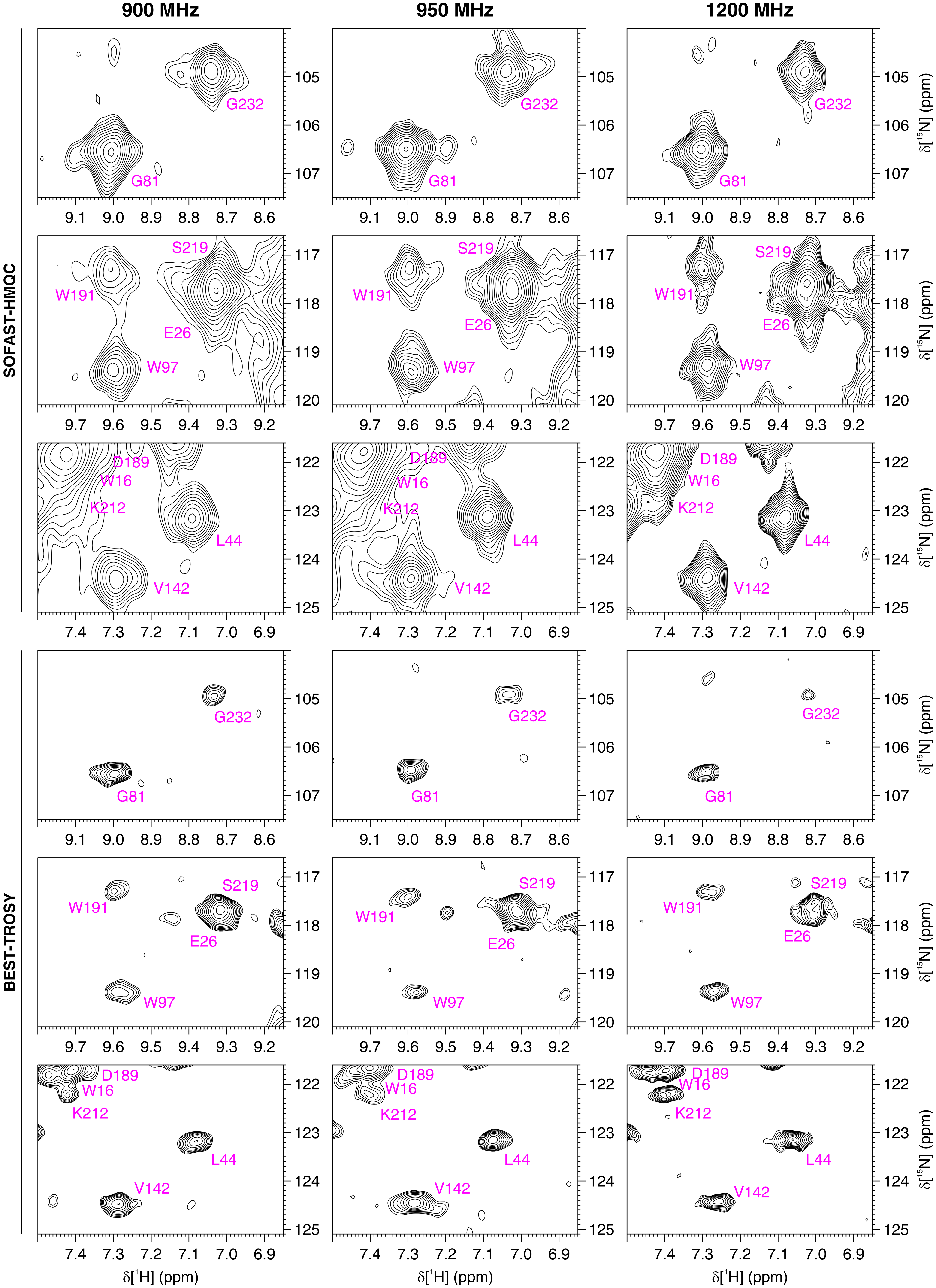
**

**Fig. S9** CA II in-cell NMR spectra. Spectral regions of ^1^H-^15^N SOFAST-HMQC (top series) and ^1^H-^15^N BEST-TROSY (bottom series) recorded at 900 MHz (left), 950 MHz (center) and 1.2 GHz (right) on cells expressing CA II. The lowest contour level was set to the noise height. Amide crosspeaks are labeled according to their residues (magenta).


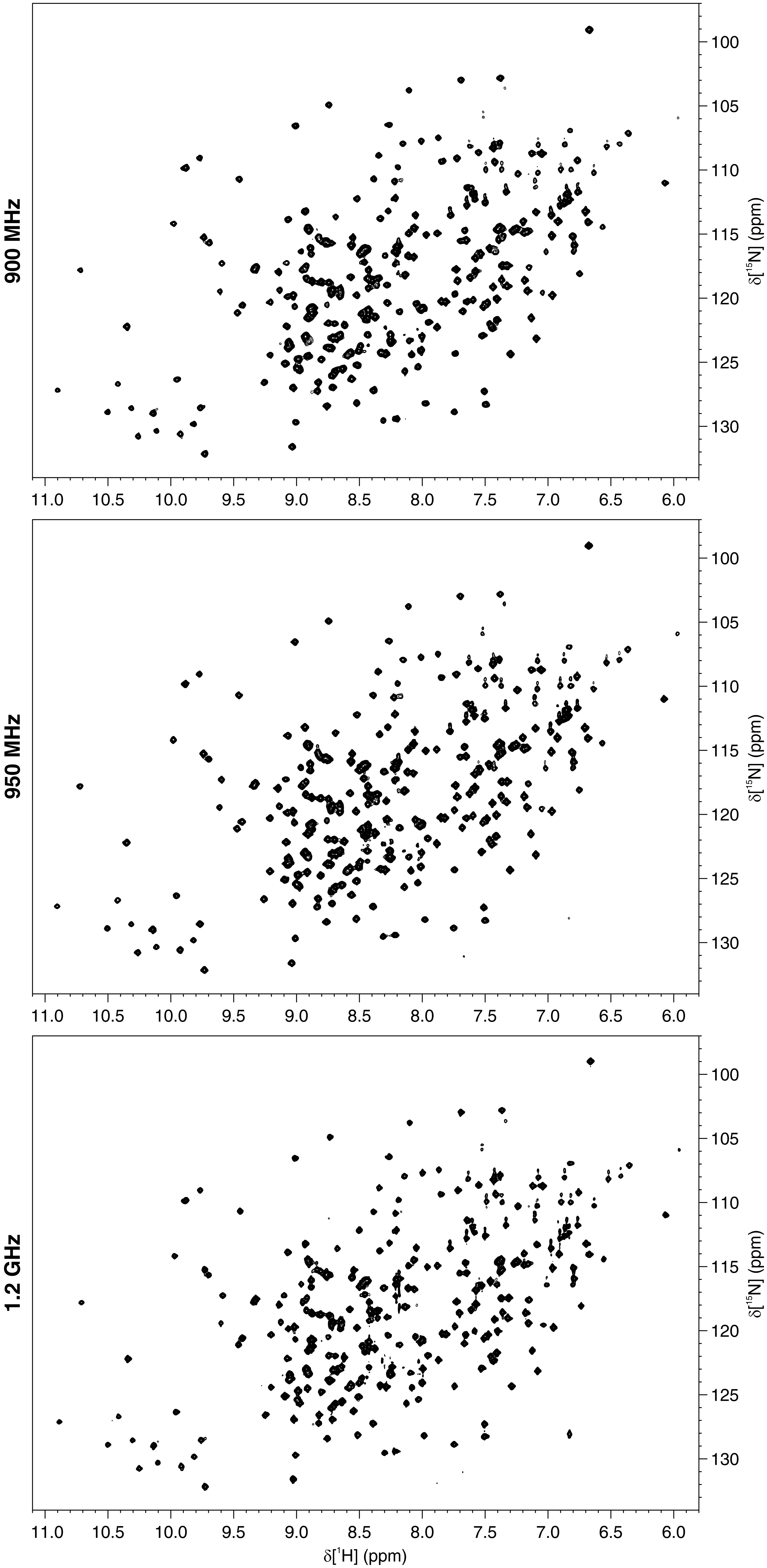


**Fig. S10** ^1^H-^15^N SOFAST-HMQC recorded at 900 MHz (top), 950 MHz (middle) and 1.2 GHz (bottom) on the lysate from cells expressing CA II. The spectra were background-subtracted (see Materials and Methods). The lowest contour level was set to 2x the noise height.


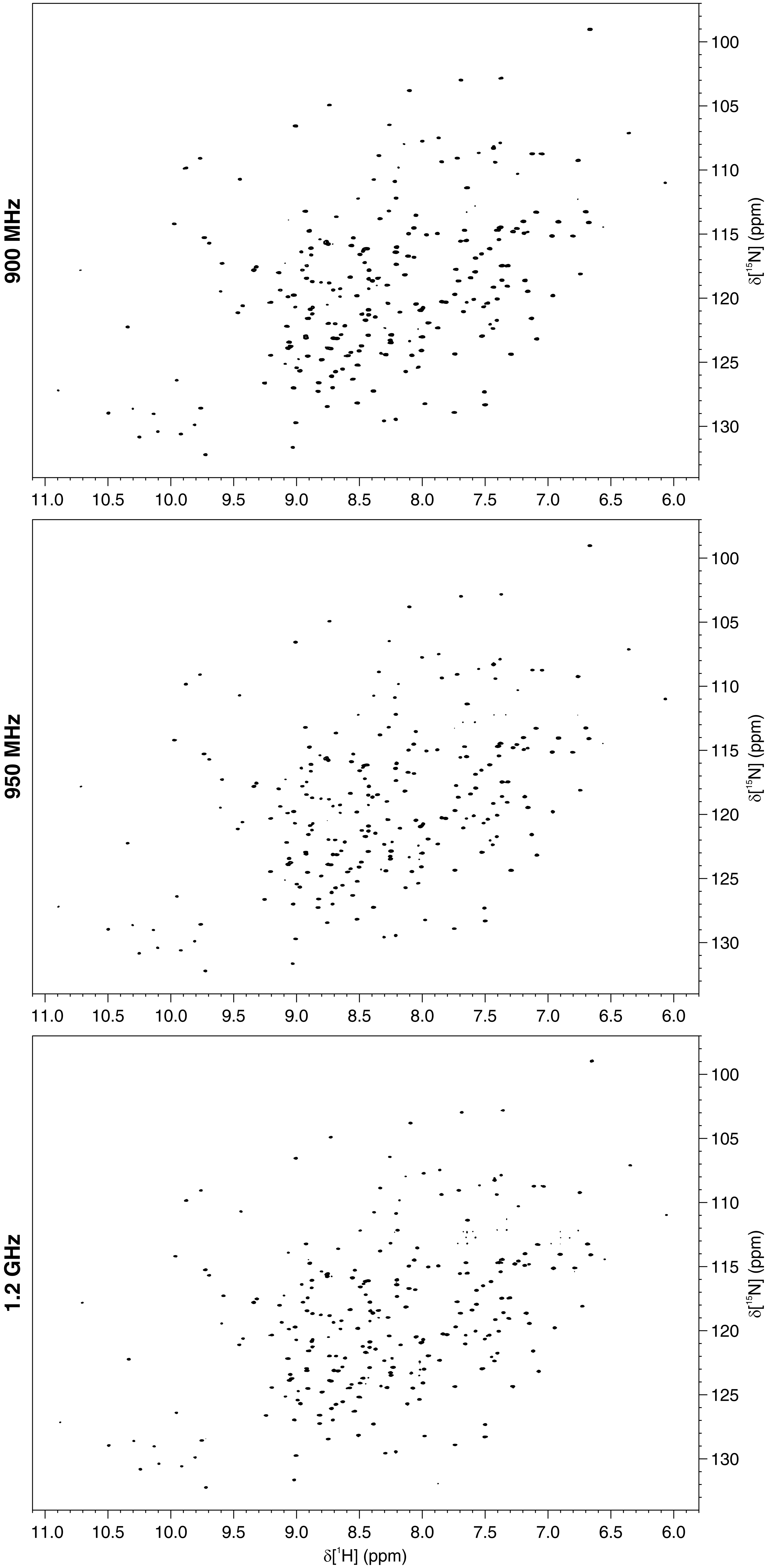


**Fig. S11** ^1^H-^15^N BEST-TROSY recorded at 900 MHz (top), 950 MHz (middle) and 1.2 GHz (bottom) on the lysate from cells expressing CA II.
The spectra were background-subtracted (see Materials and Methods). The lowest contour level was set to 2x the noise height.

**
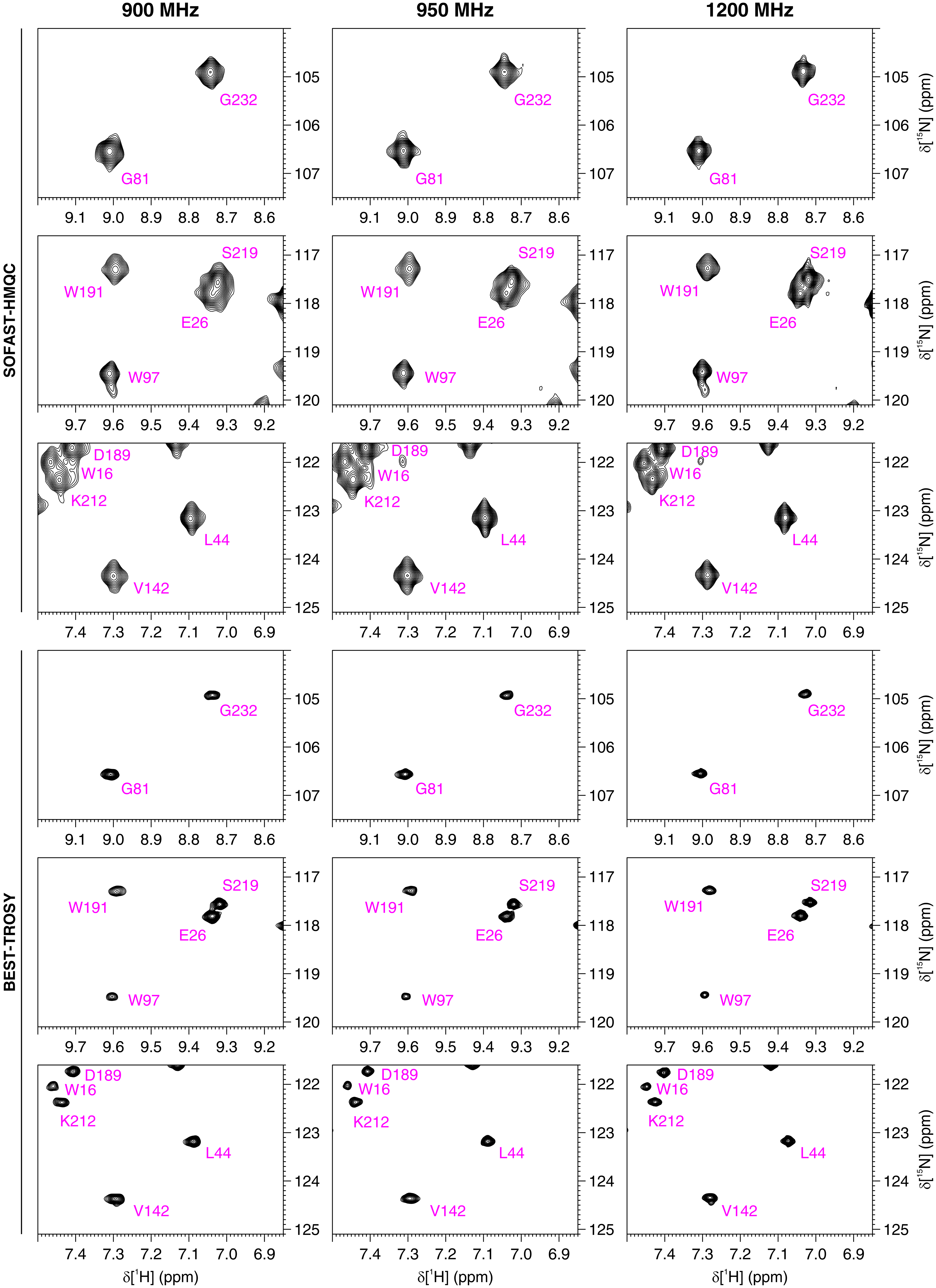
**

**Fig. S12** NMR spectra of the CA II cell lysate. Spectral regions of ^1^H-^15^N SOFAST-HMQC (top series) and ^1^H-^15^N BEST-TROSY (bottom series) recorded at 900 MHz (left), 950 MHz (center) and 1.2 GHz (right) on the lysate from cells expressing CA II. The lowest contour level was set to 2x the noise height. Amide crosspeaks are labeled according to their residues (magenta).

**
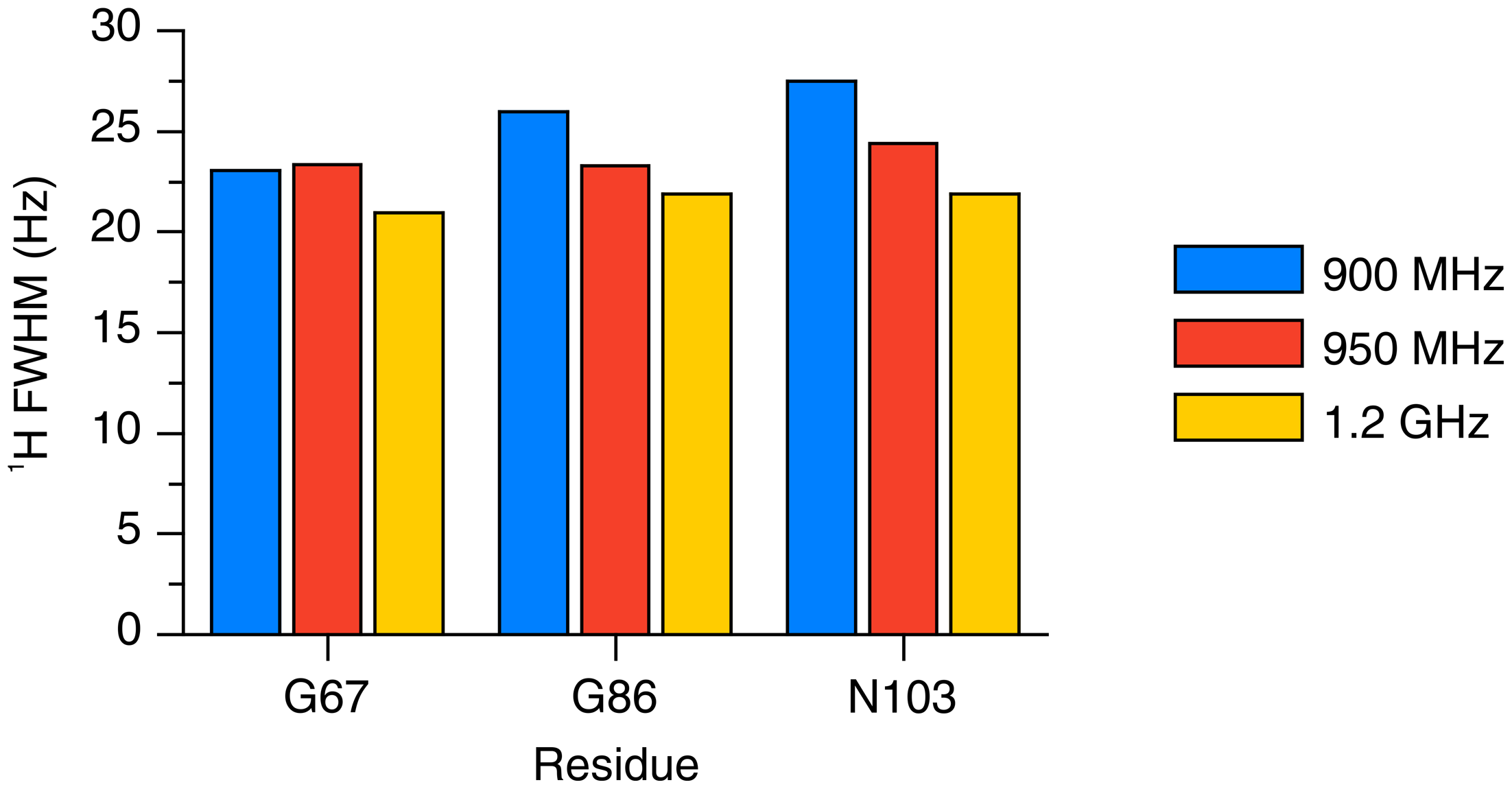
**

**Fig. S13** Linewidth analysis of α-Syn at increasing magnetic field. ^1^H FWHM (Hz) measured for three peaks in the SOFAST-HMQC spectra acquired on the cell lysate at each spectrometer. Blue: 900 MHz; red: 950 MHz; yellow: 1.2 GHz.

**Table S1** Acquisition parameters for the 2D NMR spectra recorded on cells (top) and lysates (bottom).

|  | **CELLS** | | | | | | | | | | | |
| --- | --- | --- | --- | --- | --- | --- | --- | --- | --- | --- | --- | --- |
|  | **α-synuclein** | | | | | | **carbonic anhydrase II** | | | | | |
|  | **SOFAST-HMQC** | | | **BEST-TROSY** | | | **SOFAST-HMQC** | | | **BEST-TROSY** | | |
| ^1^H Larmor freq. (MHz) | 900 | 950 | 1200 | 900 | 950 | 1200 | 900 | 950 | 1200 | 900 | 950 | 1200 |
| size of ^1^H FID | 1536 | 1600 | 2048 | 1536 | 1608 | 2080 | 1024 | 1064 | 1344 | 1152 | 1192 | 1536 |
| size of ^15^N FID | 256 | 272 | 344 | 480 | 512 | 640 | 108 | 114 | 144 | 192 | 200 | 256 |
| number of scans | 32 | 32 | 24 | 16 | 16 | 16 | 32 | 32 | 32 | 48 | 48 | 32 |
| ^1^H spectral width (ppm) | 12.0725 | 11.9955 | 12.2462 | 12.0725 | 11.9955 | 12.2462 | 16.3334 | 15.994 | 16.0142 | 16.3334 | 15.994 | 16.0142 |
| ^15^N spectral width (ppm) | 32.0462 | 32.0519 | 32.0986 | 32.0462 | 32.0519 | 32.0986 | 50.0448 | 50.1683 | 50.1051 | 50.0448 | 50.1683 | 50.105 |
| ^1^H acquisition time (ms) | 70.656 | 70.1867 | 69.632 | 70.656 | 70.5376 | 70.72 | 34.816 | 35.0056 | 34.944 | 39.168 | 39.2168 | 39.936 |
| ^15^N acquisition time (ms) | 43.776 | 44.064 | 44.032 | 82.08 | 82.944 | 81.92 | 11.826 | 11.799 | 11.808 | 21.024 | 20.7 | 20.992 |
| duration (s) | 3337 | 3401 | 3354 | 3364 | 3416 | 4476 | 1531 | 1541 | 1720 | 3356 | 3672 | 3000 |
|  |  |  |  |  |  |  |  |  |  |  |  |  |
|  | **LYSATES** | | | | | | | | | | | |
|  | **α-synuclein** | | | | | | **carbonic anhydrase II** | | | | | |
|  | **SOFAST-HMQC** | | | **BEST-TROSY** | | | **SOFAST-HMQC** | | | **BEST-TROSY** | | |
| ^1^H Larmor freq. (MHz) | 900 | 950 | 1200 | 900 | 950 | 1200 | 900 | 950 | 1200 | 900 | 950 | 1200 |
| size of ^1^H FID | 2264 | 2368 | 3072 | 2264 | 2368 | 3072 | 1984 | 2048 | 2560 | 2976 | 3072 | 3872 |
| size of ^15^N FID | 480 | 504 | 640 | 864 | 912 | 1152 | 244 | 256 | 320 | 484 | 512 | 648 |
| number of scans | 8 | 8 | 4 | 8 | 8 | 4 | 16 | 16 | 16 | 16 | 16 | 16 |
| ^1^H spectral width (ppm) | 12.0725 | 11.9955 | 12.2462 | 12.0725 | 11.9955 | 12.2462 | 16.3334 | 15.994 | 16.0142 | 16.3334 | 15.994 | 16.0142 |
| ^15^N spectral width (ppm) | 32.0462 | 32.0519 | 32.0986 | 32.0462 | 32.0519 | 32.0985 | 50.0448 | 50.1683 | 50.1051 | 50.0448 | 50.168 | 50.105 |
| ^1^H acquisition time (ms) | 104.144 | 103.8763 | 104.448 | 104.144 | 103.8763 | 104.448 | 67.456 | 67.3792 | 66.56 | 101.184 | 101.0688 | 100.672 |
| ^15^N acquisition time (ms) | 82.08 | 81.648 | 81.92 | 147.744 | 147.744 | 147.456 | 26.718 | 26.496 | 26.24 | 52.998 | 52.9922 | 53.136 |
| duration (s) | 1774 | 1776 | 1187 | 3325 | 3336 | 2211 | 1544 | 1632 | 2016 | 3327 | 3546 | 4439 |
